# A mechanochemical model recapitulates distinct vertebrate gastrulation modes

**DOI:** 10.1126/sciadv.adh8152

**Published:** 2023-12-06

**Authors:** Mattia Serra, Guillermo Serrano Nájera, Manli Chuai, Alex M. Plum, Sreejith Santhosh, Vamsi Spandan, Cornelis J. Weijer, L. Mahadevan

**Affiliations:** ^1^ Department of Physics, University of California San Diego, La Jolla, CA 92093, USA.; ^2^ Division of Cell and Developmental Biology, College of Life Sciences, University of Dundee, Dundee DD1 5EH, UK.; ^3^ School of Engineering and Applied Sciences, Harvard University, Cambridge, MA 02138, USA.; ^4^ Departments of Physics, and Organismic and Evolutionary Biology, Harvard University, Cambridge, MA 02138, USA.

## Abstract

During vertebrate gastrulation, an embryo transforms from a layer of epithelial cells into a multilayered gastrula. This process requires the coordinated movements of hundreds to tens of thousands of cells, depending on the organism. In the chick embryo, patterns of actomyosin cables spanning several cells drive coordinated tissue flows. Here, we derive a minimal theoretical framework that couples actomyosin activity to global tissue flows. Our model predicts the onset and development of gastrulation flows in normal and experimentally perturbed chick embryos, mimicking different gastrulation modes as an active stress instability. Varying initial conditions and a parameter associated with active cell ingression, our model recapitulates distinct vertebrate gastrulation morphologies, consistent with recently published experiments in the chick embryo. Altogether, our results show how changes in the patterning of critical cell behaviors associated with different force-generating mechanisms contribute to distinct vertebrate gastrulation modes via a self-organizing mechanochemical process.

## Teaser

A mechanochemical model explains self-organizing avian gastrulation flows and recapitulates other vertebrate gastrulation modes.

## INTRODUCTION

**Fig. 1.**
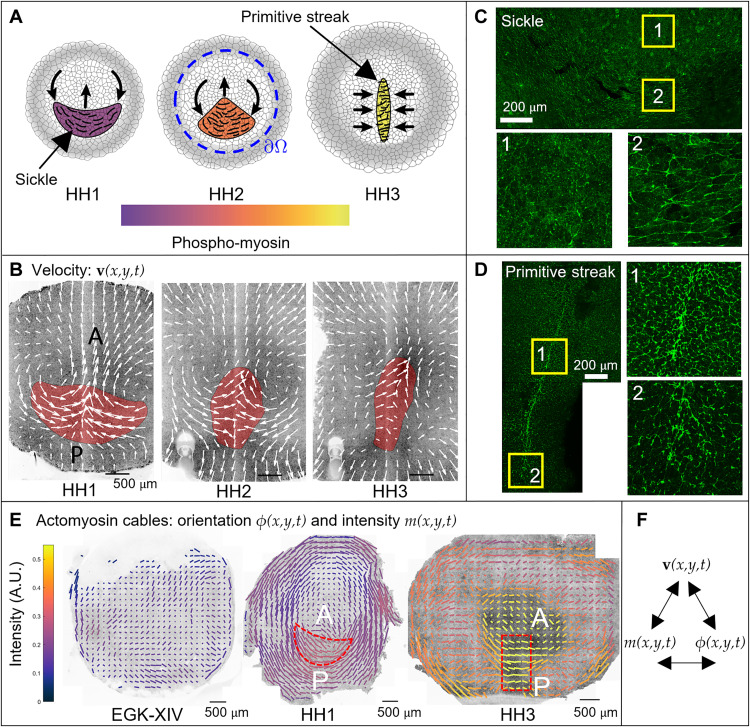
Actomyosin cables drive the tissue flows in gastrulation. (**A**) PS formation diagram. (**B**) Velocity in the chick gastrula. The convergent extension of the mesendoderm (red) generates macroscopic tissue flows on the surface of the embryo. (**C**) At HH1, the mesendoderm sickle territory is characterized by supracellular cables with active myosin (phosphorylated myosin light chain; pMLC) perpendicular to the anterior-posterior (AP) direction (see fig. S9 for details). (**D**) At HH3—12 hours after HH1—the PS is characterized by supracellular cables with high active myosin (pMLC) perpendicular to the midline. (**E**) The pattern of actomyosin cables evolves from EGK-XIV to HH3. Bar lengths represent the pMLC anisotropy and bar colors represent the measured absolute concentration. The red dashed sickle and rectangle indicate the mesendoderm precursors region and a stripe of tissue containing the PS. At HH3, the PS region has maximal pMLC intensity and maximal anisotropy perpendicular to the PS, resulting in the highest active stress along cables perpendicular to the PS. Figure S10 shows the same measured pMLC signal intensity as a scalar field over the embryo, demonstrating an overall increase in myosin activity. For experimental details, see (*4*). (**F**) Here, we construct a theoretical framework coupling velocity, actomyosin orientation, and intensity that explains observed gastrulation flows in recent experiments.

Gastrulation is a highly conserved process in the development of all vertebrate embryos (*1*), with the chick being an extensively studied model because it is easily cultured and imaged. During gastrulation, the chick transforms from a layer of epithelial cells into a layered structure of three major embryonic tissues: the ectoderm, mesoderm, and endoderm. At the moment of egg-laying, the chick embryo contains around 30,000 cells organized in a circular epiblast that will give rise to the embryo proper (EP) surrounded by a ring of extraembryonic (EE) tissue. During the first few hours of development, signals from the EE tissues and the developing hypoblast induce cells to differentiate into mesendoderm precursors in a sickle-shaped domain at the posterior edge of the epiblast (Fig. 1A). These mesendoderm precursor cells undergo directed cell intercalations that result in a contraction of the mesendoderm tissue toward its central midline, followed by an extension in the anterior direction, forming the primitive streak (PS) (Fig. 1, A and B) (*2*–*6*). In the streak, mesendoderm precursor cells undergo an epithelial-to-mesenchymal transition (EMT), followed by their individual ingression and migration into the developing embryo to form various mesodermal and endodermal structures (*7*, *8*). These cell behaviors drive embryo-scale counter-rotating tissue flows that converge at the site of PS formation (Fig. 1B) (*9*, *10*).

Three cell-scale active force-generating processes drive tissue flows: (i) the outward migration of the cells attached to the vitelline membrane at the boundary of the embryo, (ii) the active intercalation of the mesendoderm precursors generating forces in the plane of the epiblast, and (iii) the ingression of mesendoderm cells in the streak, which attracts cells toward the streak and drives the out of plane motion. The variation in tissue flows and morphologies seen across vertebrate gastrulation, ranging from fish and amphibians via reptiles to amniotes such as chicks and humans, are due to differences in embryo geometry and the relative contributions of these three force-generating processes.

A major unresolved question is which mechanisms underlie the coordination of these large-scale reproducible tissue flows during gastrulation. The onset of directional intercalations in the chick embryo correlates with the appearance of oriented chains of aligned junctions containing high levels of active myosin II motor proteins, as detected through phosphorylated myosin light chain (pMLC) (Fig. 1, A, left, and C) (*2*, *3*). These supracellular chains of aligned cell junctions with high levels of active myosin are oriented in the direction of cell intercalation, indicating the appearance of a supracellular oriented organization (planar cell polarity) of intercalating cells (Fig. 1E at HH1 stage and fig. S9). As the PS extends, supracellular cables of highly active myosin reorient perpendicular to the midline (Fig. 1, A, right, D, and E at HH3). Figure 1E shows the orientation of actomyosin cables (bar orientation), the extent of alignment of multicellular cables (bar length) quantified by the pMLC anisotropy, and the active myosin strength quantified by pMLC intensity (bar color) on the whole embryo at EGK-XIV, HH1, and HH3. The pMLC anisotropy is quantified from the asymmetry of the Fourier power spectrum (*11*), calculated in a tiling pattern over the embryo, while myosin intensity is calculated as average intensity in the same tiles. For experimental details, see the recently published paper (*4*). The panels show a high increase of active myosin over time (see also fig. S10), and at HH3, the PS region has maximal pMLC intensity and maximal anisotropy perpendicular to the PS.

These actomyosin cables, first observed in the directional cell intercalation underlying germ-band extension in fruit fly embryogenesis (*12*, *13*), were thought to arise from signaling in the anterior-posterior patterning system (*14*, *15*). Recent work (*16*–*19*) suggests instead that actomyosin cables could self-organize in a tension-dependent manner, leading to spontaneous large-scale orientation during germ-band extension. Consistent with this, it is now well established that cytoskeletal actin dynamics, myosin activity, and adhesion are mechanosensitive (*20*, *21*), e.g., contraction of a given junction will increase the tension of neighboring junctions, which in turn activates the myosin assembly and lowers the dissociation rate of active myosin in those junctions through a catch-bond mechanism (*22*–*24*). This positive feedback mechanism could then result in the formation of supracellular chains of myosin-enriched junctions at the cellular level that drive coordinated supracellular directional intercalations, organized tissue flows, and PS formation (*5*). In the chick, the early embryonic layer can be abstracted as a thin two-dimensional (2D) fluid in which myosin activity generates active stresses that induce multicellular flows. Interfering with myosin activity correlates with the failure of PS formation (*2*, *4*). But what are the essential mechanisms sufficient to generate supracellular coordination?

To probe the conditions for the self-organization of actomyosin cables during development and correlate these with observed tissue flows, we need a quantitative predictive model. Theoretical approaches include statistical and continuum theories for active matter (*25*), vertex models (*26*–*28*), and constitutive laws for epithelia (*29*), with their relative strengths and weaknesses summarized in a recent review (*30*). Theoretical work has also started to elucidate the interplay of signaling, mechanics, and geometrical constraints (*31*–*40*) reviewed in (*41*). Here, we complement these studies and devise a continuum model that is specific enough to capture the essence of the distinct gastrulation modes we observed in wild-type and experimentally perturbed chick embryos while being general enough to apply to other morphogenetic processes.

### Mathematical framework for gastrulation flows

#### Predictive model linking mechanochemical activity and tissue flows

Minimally, a planar mechanochemical predictive model of PS formation during gastrulation should couple three coarse-grained fields (Fig. 1F): the tissue velocity field **v**(*x*, *y*, *t*) = [*u*(*x*, *y*, *t*), *v*(*x*, *y*, *t*)]^⊤^, the active stress intensity *m*(*x*, *y*, *t*) arising from myosin activity, and the average cable orientation ϕ(*x*, *y*, *t*). In the limit of slow, viscous flows associated with morphogenesis, we can neglect inertia so that the local force balance reads ∇ · **σ***_T_* = **0**, where the total stress **σ***_T_* = **σ***_V_* + **σ***_A_* is the sum of the viscous and active stresses. The viscous stress is **σ***_V_* = −*p***I** + 2μ**S***_s_*, where *p* is the pressure, **I** is the identity matrix, μ is shear viscosity, and **S***_s_* = (∇ **v** + ∇ **v**^⊤^ − (∇ · **v**)**I**)/2) is the deviatoric rate-of-strain tensor. The active stress associated with actomyosin cables is **σ***_A_* = *m*(**B** − **I**/2), with the components of **B** given by *B*_11_ = cos^2^ϕ, *B*_12_ = *B*_21_ = sin 2ϕ/2, *B*_22_ = sin^2^ϕ.

We model the sheet-like embryo as a two-dimensional compressible fluid to accommodate cell ingression into the third dimension. This necessitates a continuity law modeling the expectation that both the passive isotropic stress and isotropic effects of myosin activity contribute to a negative divergence (or convergence) of the local velocity field. We choose a simple linear model with ∇ · **v** = *c*(−2*p* − *p*_0_*m*). Here, *c*^−1^ is the fluid bulk viscosity, −2*p* is the isotropic viscous stress, and *p*_0_*m* characterizes the effect of active cell ingression due to myosin-induced apical contraction (see sections S1.1 and S1.4.2 for a detailed description and the biophysical implications of *p*_0_). This continuity law enables us to express the local pressure *p* as a function of ∇ · **v** and *m*. Then, we may write the equations for local momentum balance for the planar velocity field **v**(*x*, *y*, *t*), along with the evolution equations for the orientation ϕ(*x*, *y*, *t*) and intensity *m*(*x*, *y*, *t*) of active stress as

$$\left\{ \;\begin{array}{cc} & \text{Force balance:}\\ & \overset{viscous}{\overbrace{\underset{shear}{\underbrace{2p_{1}\text{Δ}v}}+\underset{dilatation}{\underbrace{\nabla [\nabla \cdot v]}}}}+\overset{active}{\overbrace{2p_{1}(B\nabla m+m\nabla \cdot B)+p_{1}(p_{0}-1)\nabla m}}=0\;(1a)\\ & \text{Dynamics of active stress orientation:}\\ & \phi_{t}=\overset{advection}{\overbrace{-(v\cdot \nabla )\phi }}+\overset{rigid\;rotation}{\overbrace{\frac{\omega }{2}}}+\overset{shear-induced\;rotation}{\overbrace{p_{2}\left(\frac{u_{y}+v_{x}}{2}\cos2\phi +\frac{v_{y}-u_{x}}{2}\sin2\phi \right)}}\;(1b)\\ & \text{Dynamics of active stress intensity:}\\ & m_{t}=\overset{advection}{\overbrace{-(v\cdot \nabla )m}}+\overset{recruitment}{\overbrace{p_{3}}}\overset{dissociation}{\overbrace{-p_{4}e^{-\frac{p_{5}}{2}m}m}}\overset{saturation}{\overbrace{-p_{6}m}}+\overset{tension\;propagation\to induction\;of\;m}{\overbrace{p_{7}{\text{Δ}}_{\phi }m}}\\ & \;\;\;\;\;\;\;\;\;\;\;\;\;\;\;\;\;\;\;\;\;\;\;\;\;\;\;\;\;\;\;\;\;\;\;\;\;\;\;\;\;(1c) \end{array}\right.$$

Here, we have nondimensionalized our model using a characteristic length scale *x_c_*, speed *u_c_*, and viscous shear stress μ*u_c_*/*x_c_*. Equation 1a denotes the balance of forces, wherein the first and second terms are the passive, viscous forces due to shear and dilatational deformations. The last three terms are a consequence of active forces induced by inhomogeneities in the active stress intensity generated by actomyosin cables, while *p*_1_ = μ*c* is the ratio of the shear viscosity to the bulk viscosity (see section S1 for the derivation of these equations and fig. S11 for the effect of a higher ratio). Equation 1b describes the average orientational dynamics of active stress, dictated by the orientation of actomyosin cables which evolve like material fibers, advected by the flow, and rotated by vorticity ω and shear (*42*). Shear-induced rotation rates are modulated by the nondimensional parameter *p*_2_ ≈ 1 for elongated fibers.

**Fig. 2.**
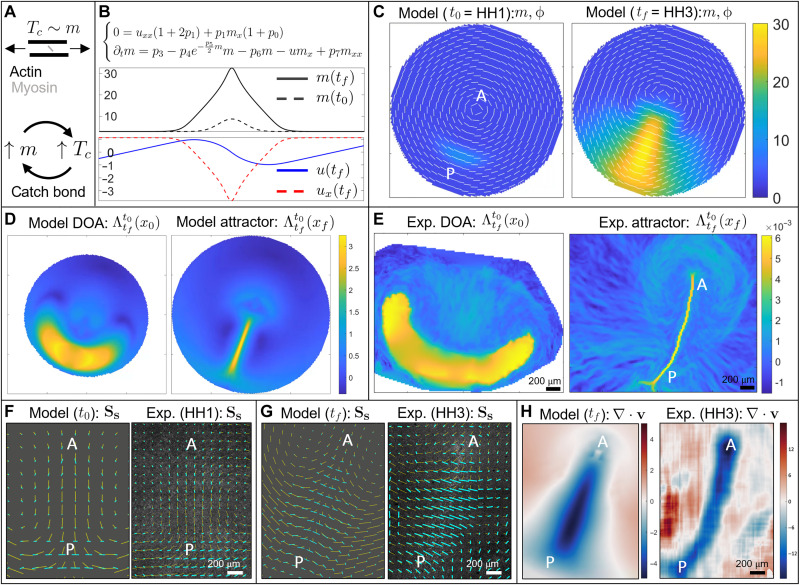
Dynamics of wild-type gastrulation in the chick embryo. (**A**) In the gastrulation timescale, the tension in the actomyosin cables is maintained by their active stress intensity *T_c_* ≈ 〈**e**, **σ***_A_***e**〉 = *m*/2 (section S1.3). High *T_c_* induces a further increase of *m* via the catch-bond mechanism, which in turn increases *T_c_*. This positive feedback process causes the instability of Eq. 1. (**B**) One-dimensional model recapitulates a focusing-type instability of active myosin, which induces a colocated velocity sink describing cell ingression at the PS. Movie S1 shows the time evolution of *m*, *u*, *u_x_*. (**C**) Initial (*t*_0_) and final (*t_f_*) distributions of *m*, ϕ for the 2D model. The instability of *m* drives cable reorientation and active myosin evolution. These, in turn, drive the tissue flows underlying the extension of the streak from posterior (P) to anterior (A). (**D**) Model-based domain of attraction (DOA) at *t*_0_ (HH1) and the attractor at *t_f_* (HH3). Movie S2 shows the time evolution of the relevant model-based **v**, *m*, ϕ, Lagrangian grid, repellers, DOAs, and attractors. (**E**) Same as (D) for the experimental velocity. Movie S3 shows the time evolution of the Lagrangian metrics for the experimental **v**. Panel (E) adapted with permission from (*44*). (**F** and **G**) Line segments indicate the velocity (yellow) and contracting eigenvector of the deviatoric rate of strain tensor (cyan) with length proportional to the contraction strength. (F) Model-based and experimental velocity deviatoric rate of strain at HH1 (*t*_0_). (G) Same as (F) at HH3 (*t_f_*). The experimental panels’ background consists of fluorescence images. (**H**) Model-based and experimental velocity divergence at HH3 (*t_f_*). Color bars in (C), (D), and (H) (left) are in nondimensional units. Color bars in (E) and (H) (right) are in 1/minutes. See section S4.6 for parameters, boundaries, and initial conditions used in Eq. 1.

Equation 1c describes the dynamics of active stress intensity. The first two terms account for cell motion (advection) and the recruitment of myosin from the cytoplasm, assumed to have a uniform constant concentration *m_n_*, at scaled rate *p*_3_ = (*t_p_*/*t_r_*)(*m_n_x_c_*)/(μ*u_c_*). Here, *t_r_* is the recruitment timescale and *t_p_* is the timescale for converting active myosin into tissue-scale active stress. This complex process involves kinetic reactions, single cells, and multicellular mechanochemical operations (section S1.3). The third term models a tension (*T_c_*)-dependent dissociation rate associated with catch-bond dynamics (Fig. 2A), which has an exponential form *p*_4_*e*^*p*_5_*T_c_*^ (*23*). Using a combination of experimental results, vertex models, and timescale arguments, in section S1.3, we show that at gastrulation (i.e., long) timescales, the tissue scale *T_c_* ≈ *m*/2. *p*_4_ = *t_p_*/*t_d_* is the ratio of *t_p_* to the dissociation timescale, and *p*_5_ = (*k_o_*μ*u_c_*)/*x_c_* is the ratio of the characteristic shear stress of the system to the characteristic bonding stress $k_{o}^{-1}$ between actin and myosin. The fourth term on the right side of the last equation ensures that active stress intensity does not accumulate without bounds. We add the simplest linear saturation term in *p*_6_*m*, where *p*_6_ = χ*t_p_* and we choose χ to set a saturation value *m*_sat_ ≈ *p*_3_/*p*_6_. The precise mechanisms and functional form that control myosin saturation are beyond the scope of our model because we are interested in a finite-time developmental interval characterized by instability rather than an asymptotic equilibrium state. This is confirmed by the sensitivity analysis (section S4.3), showing that our results are robust to changes in *p*_6_. Last, the viscoelastic behavior of the cables also results in the propagation of active stress intensity along the cable orientations, making the dynamics of active stress intensity non–cell autonomous (*37*). At the tissue scale, this results in directional diffusion of active stress intensity Δ_ϕ_*m* along the local cable orientation ϕ in Eq. 1c (see section S1.3 for derivation and section S3 for visualization). *p*_7_ = ξ/(*x_c_u_c_*) is the ratio between the transport of active stress intensity via advection and induction of active stress intensity via cable tension propagation, where ξ captures the tissue-scale effect of tension propagation.

We note that the force balance (Eq. 1a) to determine **v**(*x*, *y*, *t*) is elliptic, i.e., **v**(*x*, *y*, *t*) at a particular location depends on **v**(*x*, *y*, *t*) throughout the embryo. Because **v**(*x*, *y*, *t*) affects *m*(*x*, *y*, *t*), ϕ(*x*, *y*, *t*) (Eqs. 1b to 1c), the local active stress dynamics also depend on **v** in the whole embryo. In addition, the directional diffusion in the dynamics of the myosin field *m* makes the model non–cell autonomous even in Lagrangian coordinates, i.e., along cell trajectories. Since the chick embryo is roughly planar and circular, we solve Eq. 1 in polar coordinates by considering a circular spatial domain with boundary ∂Ω (Fig. 1A) that encloses the embryonic area and a small fraction of the EE region. This allows us to keep the size of the domain approximately fixed, as the embryonic area remains constant during gastrulation.

To complete the formulation of the problem, we need boundary and initial conditions. We impose a velocity normal to the boundary to represent epiboly and no flux for *m*, ϕ, as there is no experimental evidence of sources, sinks, or prescribed values of *m*, ϕ close to the EP-EE boundary. For details on model derivation, see sections S1.1 to S1.3. For nondimensionalization, see section S1.4. For the model in polar coordinates, see section S1.5. For the numerical scheme, see section S2. For parameter selection and sensitivity analysis, see section S4. The tension-dependent, non–cell-autonomous dynamics of actomyosin cables and the coupling of tissue compressibility to tissue stress and active myosin are two key aspects that our framework adds to the growing evidence accounting for feedback between mechanical and biochemical processes in development.

### Quantification of spatiotemporal morphogenetic flows

To complement the theoretical model (Eq. 1) for the spatiotemporal patterning of actomyosin activity and flow, we also need a properly invariant approach to quantify the resulting 2D spatiotemporal flows and compare our model results with experiments. Any framework to analyze spatiotemporal trajectories in morphogenesis requires a self-consistent description of cell motion that is independent of the choice of reference frame. This frame-invariant property, called objectivity (*43*), is a fundamental requirement that ensures that the description of deforming biological tissue is independent of the coordinate frames we choose to describe its motion. Nonobjective metrics, such as cell velocities, will yield different (inconsistent) results if they are described from a frame moving with a drifting embryo or a fixed lab frame [see, e.g., figure 1 in (*44*)].

By contrast, Lagrangian coherent structures that lead to the notion of dynamic morphoskeletons (DMs) (*44*) are objective. The DM is based on a Lagrangian description (fig. S8A) of tissue deformation captured by finite-time Lyapunov exponents (FTLE), which combine local and global mechanisms along cell trajectories. The DM consists of attractors and repellers toward which cells converge or diverge over a specific time interval *T* = *t_f_* − *t*_0_. Repellers are marked by high values of the forward FTLE ${\text{Λ}}_{t_{0}}^{t_{f}}(x_{0})$; attractors are marked by high values of backward FTLE ${\text{Λ}}_{t_{f}}^{t_{0}}(x_{f})$ and their domain of attraction (DOA) by high values of the backward FTLE displayed on the initial cell positions ${\text{Λ}}_{t_{f}}^{t_{0}}(x_{0})$ (see section S6 for details and fig. S8B for an illustration). The DM reveals the organizers of spatiotemporal trajectories and is robust to noise (*44*), hence it is ideal for quantifying morphogenesis and comparing models with experiments.

## RESULTS

### Insights from the 1D model

To gain intuition, we first analyze the 1D version of Eq. 1 modeling the dynamics perpendicular to AP, as summarized in Fig. 2B (section S1.4.3). Linear stability analysis of uniform equilibria of *m* reveals that the lower equilibrium is linearly stable, the intermediate equilibrium is linearly unstable, and the higher equilibrium—set by the linear saturation term—is linearly stable (fig. S3). Initializing the nonlinear system with a Gaussian perturbation *m*(**x**, *t*_0_) near the unstable equilibrium (mimicking the onset of actomyosin cables in the sickle as in Fig. 1, A, C, and E), we see that our model develops a focusing-type instability which increases *m*(**x**, *t_f_*) while generating a highly compressive region *u_x_*(*t_f_*) ≪ 0, representing cell ingression at the streak. This process shows that a region with higher initial active stress intensity provided by higher myosin induces more tension along actomyosin cables (Fig. 2A), which results in the recruitment of more active myosin, thus increasing the active stress intensity via the positive feedback mechanism embodied in Eq. 1c. Movie S1 shows the time evolution of the relevant fields. While this simplified 1D model neglects cable orientations, it accounts for the important roles of both the catch-bond dynamics coupled with the flow field, and our effective constitutive law, both of which carry over to the 2D model. However, without accounting for the dynamics of actomyosin cable reorientation, this simple 1D model cannot reproduce the 2D patterns of convergent extension and vortical flows observed in experiments.

### The 2D model predicts epiblast tissue flows during chick gastrulation

We initialize the 2D model with *m*(**x**, *t*_0_) consisting of a curved Gaussian perturbation to the unstable equilibrium of *m*, mimicking the sickle-shaped region of mesendoderm precursors, and ϕ(**x**, *t*_0_) in the azimuthal direction (Fig. 2C), consistent with experiments (Fig. 1E at HH1) (*4*). See fig. S12 for the effects of varying the initial extent of this region. We note that understanding what sets these initial conditions is beyond the scope of this work. The instability of *m* drives both the flow velocity and cable orientations shown in Fig. 2C and movie S2, reproducing the typical flow patterns observed in wild-type experiments. This instability mechanism is consistent with experimental observations of increasing pMLC between HH1 and HH3 (Fig. 1E and fig. S10) (*4*). A closer look at *m*, ϕ shows that while at *t*_0_ the active stress creates convergence perpendicular to the PS in the posterior, at later times, the active stress is dominant close to the PS (Fig. 2C), consistent with experiments. Figure 1E at HH3 quantitatively shows that pMLC has the highest intensity and pMLC anisotropy perpendicular to the PS, resulting in the highest active stress in the PS region, as predicted by Fig. 2C.

We now deploy the DM to quantify whether the Eulerian fields predicted by Eq. 1 at different times will properly integrate along cell paths, reproducing the observed morphogenetic features. In Fig. 2 (D and E), we show the DOA (left) and the attractor (right), corresponding to the largest time *T* in both the model and the experimental **v**. The attractor marks the formed PS at HH3, while the DOA marks the initial (HH1) position of cells that will end up in the PS. Movie S2 shows the time evolution of the model-based **v**, *m*, ϕ, Lagrangian grid, repellers, DOAs, and attractors as *T* increases, while movie S3 shows the same Lagrangian quantities obtained from the experimental velocity field. Consistent with our model, figure 1 (A to G) and movie S2 of (*4*) show bright-field images and gene expression patterns that indicate the mesendoderm territory.

As an additional model-experiment comparison, we consider the objective Eulerian quantities **S**_**s**_ and ∇ · **v**. The cyan direction field in Fig. 2 (F and G) shows the contracting eigenvector field with the bar length proportional to the contraction strength (i.e., the corresponding eigenvalue) of the deviatoric rate of strain tensor **S**_**s**_ at HH1 and HH3. The contracting direction of **S**_**s**_ and its strength are a proxy of cell intercalation, consistent with dedicated intercalation analysis in figure 4 of (*4*). These results show that at HH1 (Fig. 2F), intercalation is perpendicular to the AP direction and dominant in the sickle-shaped domain at the posterior edge of the epiblast, while at HH3 (Fig. 2G) it is perpendicular to the entire PS. At HH3, the PS region is characterized by high cell ingression, resulting in highly negative planar divergence ∇ · **v**, while the rest of the embryo has positive divergence to ensure embryonic area homeostasis. Our model predicts these results in Fig. 2H (left), consistent with experiments in Fig. 2H (right). This match of the model and experiment at the final time (HH3) supports further our simple continuity law ∇ · **v** = *c*(−2*p* − *p*_0_*m*).

However, we emphasize that matching Eulerian snapshots of **S**_**s**_ and ∇ · **v** between the model and experiments is not sufficient, as it is their cumulative effect along cell trajectories that quantifies morphogenesis (*44*). This Lagrangian sum differs from a sum at a fixed Eulerian location [as explained in detail, e.g., in section S2C of (*44*)]. For example, the DOA in Fig. 2 (D and E) marks the initial (HH1) position of cells that will eventually ingress in the PS by HH3, which results from the Lagrangian sum of ∇ · **v** along cell trajectories. In section S4, we discuss the selection of parameters, boundaries, and initial conditions using experiments and numerical simulations and show with an extensive sensitivity analysis (movies S14 to S27) that our results are robust to parameter variations.

### Model-experiment comparison of active forces

**Fig. 3.**
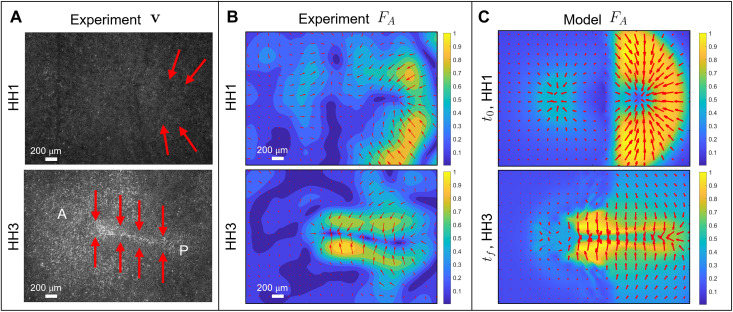
Active forces in the chick embryo. (**A**) Experimental fluorescence images at HH1 and HH3. A and P mark the anterior-posterior direction, and red arrows illustrate the dominant active force **F_A_** distribution from (**B**). At HH1, **F_A_** is dominant in the posterior embryonic region perpendicular to the AP axis. At HH3, **F_A_** is perpendicular to the PS throughout the AP axis. These **F_A_** configurations are consistent with the corresponding distribution of supracellular actomyosin cables in Fig. 1E. Dimensions are 1300 μm × 1900 μm. (B) and (**C**) Magnitude and direction of forces at HH1 (top) and HH3 (bottom). Magnitudes are normalized by the maximal spatial force. (B) We compute **F_A_** from the general force balance for highly viscous active flows **F_A_ + F_V_** = 0 (cf. Eq. 1a), which gives **F_A_** = −(**∇**[**∇** **· *v***] + 2*p*_1_**Δ*v***), where the right-hand side can be computed using experimental velocities. We use an averaging filter in space (window size ≈25 μm) and time (window size ≈15 min) on the PIV velocities and compute velocity derivatives using finite differencing. (C) **F_A_** is from Eq. 1a. We note that the model **F_A_** at HH3 arises from the dynamical variables evolved by Eq. 1 over the entire gastrulation period. They closely predict the corresponding inferred experimental forces [(B) bottom], further validating our model.

Figure 1E shows that active stress is dominant in the posterior sickle-shaped domain at HH1, while at HH3, it is dominant in the PS region, consistent with our model predictions in Fig. 2C. To support this finding further, Fig. 3 compares the active forces **F_A_** inferred indirectly from the Particle Image Velocimetry (PIV) velocities with those generated by Eq. 1. To infer the experimental **F_A_**, we use the general force balance for highly viscous active flows (as in Eq. 1a) **F_A_** + **F_V_** = 0, where **F_V_** denotes the viscous, passive force. This implies that **F_A_** = −**F_V_** = −(**∇**[**∇ · *v***] + 2*p*_1_**Δ*v***), where the right-hand side can be evaluated from experimental velocities. Figure 3A outlines the experimental distribution of active force **F_A_** from Fig. 3B over the corresponding fluorescence images.

At HH1, PIV-inferred active forces (Fig. 3B, top) are higher in the posterior sickle-shaped domain and point toward the AP axis, as predicted by our model (Fig. 3C, top). The model-based force is symmetric with respect to the AP direction. The asymmetry of the experimentally inferred active force has no effect on gastrulation except for slightly bending the PS to the left (Fig. 2E). At HH3, PIV-inferred active forces are dominant and perpendicular to the elongated PS region (Fig. 3B, bottom), as predicted by our model (Fig. 3C, bottom). This experimental evidence, consistent with the distribution pMLC in Fig. 1E and the corresponding model predictions (Figs. 2C and 3C), demonstrates that at least in the chick, active forces are not restricted to a ring-shaped structure at the EP-EE boundary, as suggested to be the case in the quail (*3*).

### The model predicts perturbations

To further test our model predictions, we move beyond gastrulation in wild-type avian embryos and introduce four different perturbations that modify either the initial state of the embryonic pattern (i.e., region of mesendoderm precursors) characterized by high phosphorylated myosin and hence active stress intensity *m*(**x**, *t*_0_), or the propensity for active cell ingression encoded in *p*_0_. In section S4.6, we summarize the values of *m*(**x**, *t*_0_) and *p*_0_ used to reproduce different gastrulation modes. The rest of the parameters are the same for all simulations in the paper (section S4.6 and table S2).

### Twin perturbation

**Fig. 4.**
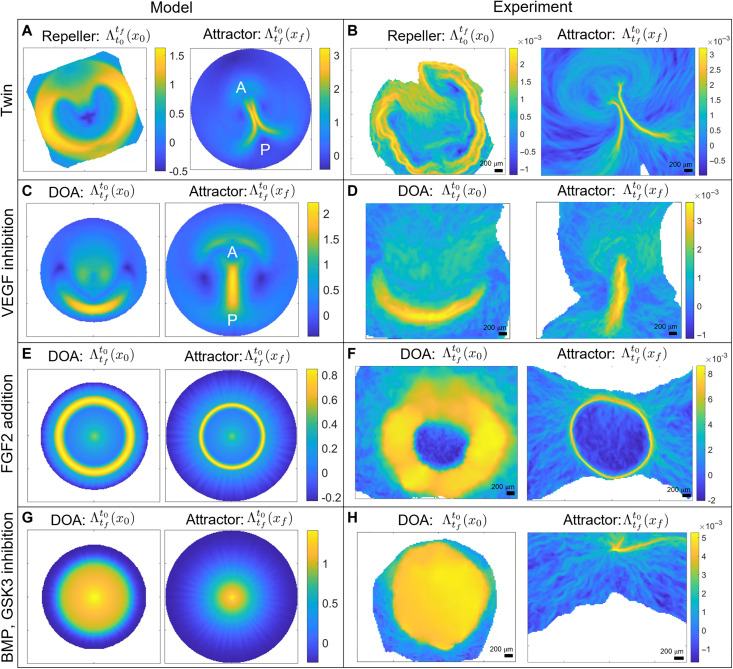
Developmental perturbations of gastrulation. Movies show the time evolution of the relevant Eulerian and Lagrangian metrics. (**A** and **B**) Spontaneous twin perturbation. Repeller and attractor for model velocities [(A), movie S4] and experimental velocities [(B), movie S5]. (**C** and **D**) VEGF (vascular epithelial growth factor) inhibition prevents cell ingression at the PS. DOA and attractor for model velocities [(C), movie S6] and experimental velocities [(D), movie S7]. (**E** and **F**) FGF2 (fibroblast growth factor 2) addition provokes a circular PS. DOA and attractor for model velocities [(E), movie S8] and experimental velocities [(F), movie S9]. (**G** and **H**) BMP (bone morphogenetic protein) and GSK3 (glycogen synthase kinase 3) inhibition induce a ring-shaped mesoderm territory at the EE-EP interface while blocking apical contraction and cell ingression. DOA and attractor for model velocities [(G), movie S11) and experimental velocities [(H), movie S12]. Color bars in experimental panels show attraction rates in 1/minutes. See section S4.6 for parameters values, boundaries, and initial conditions used in simulating Eq. 1.

Occasionally, a natural event corresponding to the spontaneous formation of twins arises when the mesendoderm precursor area is split into two regions, from which two streaks emerge. These streaks interact through their tissue flows and form complete or partially twined embryos (Fig. 4, A and B). We initialize our model as in the wild type (Fig. 2C) but add two distant Gaussians to the uniform unstable equilibrium of *m*. Movie S4 shows the model-based Eulerian fields and the induced Lagrangian metrics for increasing *T*. Figure 4A shows the model-based repeller and the attractor for the largest *T*. The circular repeller separates the EP from the EE region as well as the A-P regions of the PS, while the attractor marks the merging PSs. Figure 4B and movie S5 show the same as Fig. 4A and movie S4 for the experimental **v**. A comparison of movies S4 and S5 highlights how our model recapitulates the full morphogenetic process quantified by the DM. See fig. S13 for model predictions when PSs form opposite one another and for when one Gaussian has a greater amplitude than the other.

### Reptilian-like mode

In the second perturbation, described in detail in the recently published paper (*4*), we interfered with a signaling pathway, which resulted in a strong inhibition of cell ingression in the streak, via the application of the VEGF receptor (vascular epithelial growth factor) inhibitor axitinib (100 nM). We initialize our model as in the wild type (Fig. 2C) but set *p*_0_ = 0 as Axitinib prevents active cell ingression. Figure 4 (C and D) shows the DOA and attractor for both the model and experimental **v**, highlighting how this perturbation results in a shorter and thicker PS as well as a reduced amount of ingressed cells, consistent with the bright-field images and gene expression patterns in figure 2 (H to N) of (*4*). Movies S6 and S7 show the time evolution of the model- and experiment-based fields confirming similar DM again.

### Teleost-like mode

The third perturbation consists of experiments where FGF2 (fibroblast growth factor 2) addition to early-stage chick embryos provoked the generation of a mesoderm ring along the marginal zone (*4*). To model this case, we use the same conditions as the wild type but set *m*(**x**, *t*_0_) as a Gaussian around a fixed radius added to the unstable equilibrium [see *m*(**x**, *t*_0_) in movie S8]. Figure 4 (E and F) shows the DOA and attractor corresponding to the largest *T* from the model and experimental **v**, highlighting a sharp circular PS. Movies S8 and S9 show the Eulerian fields and the Lagrangian metrics from the model and experimental **v** for increasing *T*. In addition, Movie S10 shows a deforming Lagrangian grid overlaid on the light-sheet microscope images. These results complement those in figure 1 (H to N) of (*4*) and confirm again the predictive power of our model.

### Amphibian-like mode

In our last perturbation, we induced the formation of a ring-shaped mesoderm region at the EE-EP interface with a combination of the BMP (bone morphogenetic protein) receptor inhibitor LDN-193189 (100 nM) and the GSK3 (glycogen synthase kinase 3) inhibitor CHIR-99021 (3 μM) (*4*). This treatment also blocks apical contraction and cell ingression but has little effect on cell intercalations, resulting in the buckling of the tissue [figure 2 (A to G) and movie S8 of (*4*)]. In our model, we use the initial condition of perturbation 3 but set *p*_0_ = 0, following the same reasoning of perturbation 2. Figure 4 (G and H) shows the DOA and the attractor for the largest *T* from the model and the experimental **v**, while movies S11 and S12 display the corresponding time evolution of the Eulerian fields and Lagrangian metrics. While tissue buckling is intrinsically 3D, our model is sufficient to predict an overall planar compressive stress that causes the tissue to converge in the center. This prediction matches precisely the experimental 2D dynamics inferred by PIV velocities.

### Toward a phase space of gastrulation flow patterns

**Fig. 5.**
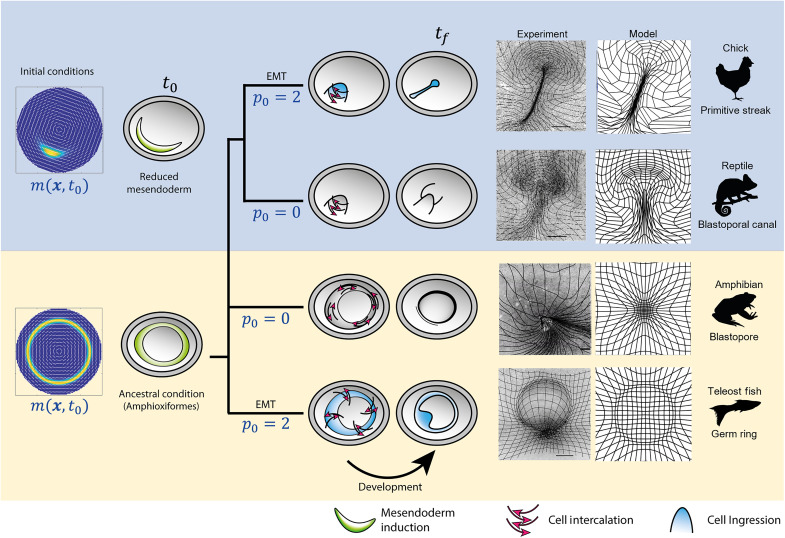
Evolutionary transitions in gastrulation patterns. A critical morphogenetic parameter *p*_0_ models the amount of EMT or active cell ingression caused by the cell’s propensity to ingress given apical myosin-induced isotropic contraction. The initial condition *m*(**x**, *t*_0_) models the extent of the mesendoderm precursor territory. Changing cell behaviors *p*_0_ and initial cell types *m*(**x**, *t*_0_) (left column), our model recapitulates the flow patterns in the phylogeny of vertebrate gastrulation from a self-organizing dynamical structure. Our model-based predicted flow patterns mimic those naturally observed in reptiles, amphibians, and fish, and are reproduced experimentally, in vivo, in the chick embryo [see also the recently published paper (*4*) for details on the experiments]. The right columns show deformed Lagrangian grids overlaying the light-sheet microscope images from perturbed chick-experiment velocities (*4*) and deformed Lagrangian grids from the predicted model velocity for each gastrulation mode. Scale bars, 500 μm.

To go beyond studying the gastrulation modes in wild-type avian embryos exemplified by the chick, we used chemical inhibitors to uncouple cell intercalation and ingression and perturb the initial patterning and function of the prospective mesendoderm, as described in our recently published experimental paper (*4*). The current paper shows that we can recapitulate these phylogenetic variations in the morphodynamics of vertebrate gastrulation using a mechanochemical model that couples the dynamics of actomyosin cables to large-scale tissue flow patterns. By changing the cell’s propensity to undergo active ingression from apical isotropic myosin contraction, characterized by the parameter *p*_0_, together with the initial prospective mesendoderm, described by *m*(**x**, *t*_0_) (Fig. 5, left), we can predict distinct gastrulation flows that resemble those naturally observed in other vertebrates, consistent with in vivo experiments in the chick embryo (Fig. 5, center right). Specifically, the PS structures in Fig. 4 (C and D) resemble the blastoporal canal observed in reptilian gastrulation (Fig. 5, second row), the circular streak structure (Fig. 4, E and F) resembles the germ ring of teleost fish gastrulation (Fig. 5, last row), while the buckling tissue in Fig. 4 (G and H) mimics the tissue organization and flow during the closing blastopore in amphibians (Fig. 5, third row). We highlight that our biophysical model is sufficient to explain both the wild-type and manipulated gastrulation flows in the chick embryo, which show remarkable similarities to the gastrulation patterns naturally observed in other species. Whether our framework can explain (at least partially) the gastrulation flows in different vertebrates is a separate biological question that requires additional work (*45*).

## DISCUSSION

In this study, we have investigated the self-organizing principles of gastrulation flows in wild-type and experimentally perturbed chick embryos. Using in vivo experiments and biophysical modeling, we have provided evidence that gastrulation movements arise from the coupled dynamics of tissue flows and mechanosensitive actomyosin activity (Eq. 1). Actomyosin activity generates active stresses, which drive tissue flows and tension. Tissue flows and tension, in turn, modulate actomyosin (or active stress) dynamics with mechanochemical feedback. Specifically, the actomyosin dissociation rate depends on the tissue-scale tension via a catch-bond mechanism, while tissue flows modulate actomyosin cable orientation. Because of the induction of active stress intensity via tension propagation, our model is non–cell-autonomous even in Lagrangian coordinates that follow cell trajectories. Our framework shows that gastrulation flows follow from an active stress instability (Fig. 2), consistent with experiments (Fig. 1 and fig. S10).

In particular, providing only the initial conditions for the actomyosin cables at the onset of chick gastrulation and the boundary conditions modeling the epiboly motion of EE cells, our model predicts subsequent gastrulation stages over 12 hours (Figs. 2 and 4 and movies S2 to S12). This allows us to recapitulate the observed dynamics of actomyosin cable reorientation, as well as the redistribution of actomyosin levels (Fig. 1E and fig. S10), the observed active forces, and the large-scale tissue motion. The experimental distribution of actomyosin cables (Fig. 1E) and the active forces inferred from PIV velocities (Fig. 3) show that in chick, active forces are not organized in a ring-shaped structure at the boundary between embryonic and EE regions, as suggested to be the case in the quail (*3*). In fact, at late gastrulation stages, active forces dominate in the PS region at the embryo’s midline (Fig. 3). While Saadaoui *et al.* (*3*) and Caldarelli *et al.* (*46*) directly prescribe active forces on a tensile ring located at the boundary between the EP and EE area, our active force distribution evolves spontaneously in space and time through Eq. 1, leading to a self-organized pattern consistent with experiments (Figs. 1E and 3B). Furthermore, our framework predicts the velocity divergence and other dynamical fields (Fig. 2 and movies S2 to S12) from the underlying self-organizing dynamics in contrast to (*3*, *46*) that fits spatiotemporal experimental velocity divergence fields into the model. A detailed comparison with the tensile-ring model is given in section S5.

By changing a nondimensional parameter modeling active cell ingression (cell behavior)—i.e., the cell’s propensity to ingress due to high myosin-induced apical contraction—together with the initial distribution of active myosin as a proxy of prospective mesendoderm tissue (cell type), our model recapitulates distinct gastrulation flows that mimic those observed in fish, amphibians, and reptiles, consistent with experiment in vivo in the chick embryo (Fig. 5). In the recently published paper (*4*), we used drugs to modulate the ability for active cell ingression and the initial extent of the prospective mesendoderm and showed that the resulting gastrulation flows mirror those naturally observed in reptiles, amphibians, and fish in a single organism, the chick embryo, consistent with our model predictions. Additional or alternative driving forces may be at play in these other species (*45*). For example, studies of amphibian gastrulation in Xenopus have suggested that convergent thickening serves as an additional driving force during blastopore closure (*47*–*49*). Studies of teleost fish gastrulation in zebrafish have suggested that active directed cell migration of mesendoderm precursors plays an important role in their internalization (*50*, *51*). Therefore, while the driving forces we have modeled are sufficient to generate flows in chick that mirror gastrulation in these other species, the exact gastrulation mechanisms in other vertebrates likely involve additional forces, not yet directly captured in our model.

To estimate the scalar, time-independent nondimensional parameters in our model, we use a combination of mechanistic arguments, experiments, and numerical simulations. We also perform an extensive sensitivity analysis showing the robustness of our results to parameter variations (section S4 and movies S14 to S27). Although our model does not account for 3D effects, the multilayered structure of EE tissue, different cell types, and the overall size and shape of embryos, we capture the essential differences in the modes of tissue flow in a range of vertebrate gastrulation. The consistency of our predictions with in vivo experiments in the chick embryo shows that a relatively small number of changes in critical cell behaviors associated with different force-generating processes contribute to the outcome of distinct vertebrate gastrulation modes via a self-organizing mechanism. The simplicity of the perturbations suggests that these divergent gastrulation morphologies might be relatively easily evolvable. A natural next step is to account for the above limitations, starting from different embryo sizes and geometries, modeling 3D effects, and explicitly accounting for distinct cell types to link tissue flows and morphogenesis with cell differentiation.

Linking the gene expression patterns to the myosin patterns and cell flows is an important step to fully understand the early stages of development. In principle, we already have the tools to map gene expression patterns on the cell flows using the Lagrangian DM framework (*44*). This mapping will allow us to couple the deformation of the gene expression domains responsible for mesendoderm induction and maintenance while ensuring further mesoderm and endoderm specification and spatial organization. These processes likely result in further changes in stress generation and mechanical feedback, completing the current theoretical framework of early chick development.

## MATERIALS AND METHODS

For details on the acquisition and processing of the experimental data used in this work, please see the material and methods section of our accompanying paper (*4*). A detailed description of the numerical schemes used to solve our model (Eq. 1) is in section S2. The numerical scheme to compute the DMs from experimental and modeled velocity fields is in section S6.

## Supplementary Materials

### This PDF file includes:

Supplementary Text Figs. S1 to S13 Tables S1 to S3 Legends for movies S1 to S27 References
