# Supplementary material for: A mechanochemical model recapitulates distinct vertebrate gastrulation modes
Source: Sci Adv. 2023 Dec 6;9(49):eadh8152. doi: 10.1126/sciadv.adh8152 (PMC10699781; doi:10.1126/sciadv.adh8152)
Supplement: Supplementary file 1 — Supplementary Text Figs. S1 to S13 Tables S1 to S3 Legends for movies S1 to S27 References [file sciadv.adh8152_sm.pdf]

Science Advances
AAAS

Supplementary Materials for

A mechanochemical model recapitulates distinct vertebrate gastrulation modes

Mattia Serra *et al.*

Corresponding author: Mattia Serra, mserra@ucsd.edu; L. Mahadevan, lmahadev@g.harvard.edu

Sci. Adv. **9**, eadh8152 (2023)
DOI: 10.1126/sciadv.adh8152

The PDF file includes:

Supplementary Text
Figs. S1 to S13
Tables S1 to S3
Legends for movies S1 to S27
References

Other Supplementary Material for this manuscript includes the following:

Movies S1 to S27

1 Mathematical Model

We derive our model in Cartesian coordinates and then provide an equivalent reformulation in polar coordinates.

1.1 Momentum Balance and Continuity Equation

We denote the tissue velocity field $\mathbf{v}(\mathbf{x}, t) = [u(\mathbf{x}, t), v(\mathbf{x}, t)]^\top$ and its vorticity scalar by $\omega(\mathbf{x}, t)$. We recall the velocity gradient decomposition

$$\nabla\mathbf{v}(\mathbf{x}, t) = \mathbf{S}(\mathbf{x}, t) + \mathbf{W}(\mathbf{x}, t), \tag{S1}$$

with the rate-of-strain tensor $\mathbf{S}$ and the spin tensor $\mathbf{W}$ defined as

$$\mathbf{S} = \frac{1}{2}(\nabla\mathbf{v} + \nabla\mathbf{v}^\top), \quad \mathbf{W} = \frac{1}{2}(\nabla\mathbf{v} - \nabla\mathbf{v}^\top) \equiv \frac{\omega}{2}\mathbf{R}, \quad \mathbf{R} := \begin{pmatrix} 0 & -1 \\ 1 & 0 \end{pmatrix}. \tag{S2}$$

We further decompose $\mathbf{S}$ into its isotropic and deviatoric parts as

$$\mathbf{S_s} = \mathbf{S} - \mathbf{S}_I, \quad \mathbf{S}_I = \mathbf{I}\frac{1}{2}\nabla \cdot \mathbf{v}. \tag{S3}$$

The viscous, active, total stresses and total stress trace are

$$\begin{aligned} \sigma_V =& -p\mathbf{I} + 2\mu\mathbf{S}_s \\ \sigma_A =& m(\mathbf{x}, t)[\mathbf{e}(\mathbf{x}, t) \otimes \mathbf{e}(\mathbf{x}, t) - \tfrac{1}{2}\mathbf{I}], \quad \mathbf{e}(\mathbf{x}, t) = [\cos\phi(\mathbf{x}, t), \sin\phi(\mathbf{x}, t)]^\top \\ \sigma_T =& \sigma_V + \sigma_A \\ \mathrm{Tr}[\sigma_T] =& -2p, \end{aligned} \tag{S4}$$

where μ denotes the shear viscosity, m denotes the active stress intensity arising from active myosin and ϕ the orientation of the myosin cables with respect to the $x-$axis. The friction exerted by the surrounding fluid on the epiblast is assumed to be very small, therefore we ignore substrate friction in our model.

Because viscosity is high ($\mu \approx 9 \times 10^3\ Pa \cdot s$), we ignore inertial terms and write the momentum balance as

$$\begin{aligned}
\nabla \cdot \sigma_T =& \nabla \cdot \sigma_V + \nabla \cdot \sigma_A = \mathbf{0} \\
\nabla \cdot \sigma_V =& \begin{bmatrix} -p_x \\ -p_y \end{bmatrix} + \mu \begin{bmatrix} u_{yy} + u_{xx} \\ v_{xx} + v_{yy} \end{bmatrix} \\
\nabla \cdot \sigma_A =& \frac{1}{2} \begin{bmatrix} \cos 2\phi\ (2m\phi_y + m_x) + \sin 2\phi (m_y - 2m\phi_x) \\ \sin 2\phi\ (2m\phi_y + m_x) - \cos 2\phi (m_y - 2m\phi_x) \end{bmatrix} \\
\implies \begin{bmatrix} p_x \\ p_y \end{bmatrix} =& \mu \begin{bmatrix} u_{yy} + u_{xx} \\ v_{xx} + v_{yy} \end{bmatrix} + \frac{1}{2} \begin{bmatrix} \cos 2\phi\ (2m\phi_y + m_x) + \sin 2\phi (m_y - 2m\phi_x) \\ \sin 2\phi\ (2m\phi_y + m_x) - \cos 2\phi (m_y - 2m\phi_x) \end{bmatrix}.
\end{aligned} \tag{S5}$$

To close the system, we choose a simple continuity equation in which the flow divergence is proportional to the isotropic total stress (eq. (S4)) and active myosin concentration i.e.

$$\begin{aligned}
\nabla \cdot \mathbf{v} =& c(\mathrm{Tr}[\sigma_T] - p_0 m) = c(-2p - p_0 m) \\
\implies & p = -\tfrac{\nabla \cdot \mathbf{v}}{2c} - \tfrac{p_0 m}{2},
\end{aligned} \tag{S6}$$

where c has units $[\ Pa \cdot s]^{-1}$ and p_0 is a non-dimensional parameter modulating the contribution of active myosin to flow divergence. The intuition behind eq. (S6) is that regions of high isotropic compressive stress and high active myosin, inducing isotropic apical cell contraction, exhibit negative flow divergence, i.e., cell ingression or folding. The above relation then serves as a means of writing the local pressure in terms of the flow divergence and the active myosin concentration. Equation (S6) can also be derived considering the continuity equation of a two-dimensional confluent epithelium and using the above phenomenological arguments to express the source/sink terms as a function of $\nabla \cdot \mathbf{v}$ and m. Substituting eq. (S6) into eq. (S5), we obtain the resulting force balance equations describing our active continuum without pressure terms

$$\begin{aligned}
0 &= u_{xx}\frac{1+2\mu c}{2c} + u_{yy}\mu + \frac{v_{xy}}{2c} + \frac{p_0 m_x}{2} + \frac{1}{2}(\cos 2\phi\ (2m\phi_y + m_x) + \sin 2\phi (m_y - 2m\phi_x)) \\
0 &= v_{xx}\mu + v_{yy}\frac{1+2\mu c}{2c} + \frac{u_{xy}}{2c} + \frac{p_0 m_y}{2} + \frac{1}{2}(\sin 2\phi\ (2m\phi_y + m_x) - \cos 2\phi (m_y - 2m\phi_x)).
\end{aligned} \tag{S7}$$

1.2 Active Stress Orientation

We assume the evolution of actomyosin cables orientation satisfies Jeffery's equation (*42*)

$$\phi_t = -[\mathbf{v} \cdot \nabla]\phi + \tfrac{\omega}{2} + \lambda(S_{12} \cos 2\phi + \tfrac{S_{22} - S_{11}}{2} \sin 2\phi), \tag{S8}$$

which describes the direction evolution of axis-symmetric fibers under a low Reynold's number flow. In eq. (S8), $\phi_t = \frac{\partial \phi}{\partial t}$, λ is a parameter quantifying the sensitivity of actomyosin cables reorientation due to shear-induced rotations, S_{ij} denotes the entries of $\mathbf{S}$ and the first two terms represent the advection term and the rigid-body rotation rate described by half of the flow vorticity.

1.3 Active Stress Magnitude

The dynamics of active myosin on actin filaments is characterized by catch bonds (*23*), i.e., the dissociation rate of actomyosin bonds depends on the tension along the actomyosin cable T_c, and decreases exponentially for increasing tension. Laser ablation experiments demonstrate that supracellular actomyosin cables exhibit higher tension and higher levels of myosin than isolated cell junctions (*52*). We explain below our reasoning to estimate T_c.

1.3.1 Junction-level modeling

Junction-level modeling of the feedback between total tension and active stress intensity supports active stress intensity as a good proxy for cable tension. Optical manipulation of apical cell-cell junctions in drosophila and chick embryo suggest that junctions are viscoelastic (*53, 54*). Among simple viscoelastic models, measurements of junction deformation through time in chick embryos are best fit by a simple Maxwell model consisting of a spring and dashpot in series (*54*) (Figure S1A). Thus, we consider each junction as a Maxwell element in parallel with an active element generating additional active stresses (Figure S1B). Maxwellian viscoelasticity has been frequently used to model single-junction dynamics in drosophila (*35, 37, 53*). It is easy to show that the tension of a Maxwell element can be written as an elastic term with a continuously relaxing rest length (*55*). Consistent with the assumption of Maxwellian viscoelasticity and additional tension induced by myosin activity, the total junction tension is

$$T_{ij} = K(r_{ij} - l_{ij}) + m_{ij}, \tag{S9}$$

where K is a spring constant, $r_{ij} = r_j - r_i$ is the length between vertex positions, l_{ij} is a dynamic rest length at which elastic tension vanishes, and m_{ij} represents an active stress intensity due to myosin activity. The rest length relaxes to the junction length over time as

$$\frac{dl_{ij}}{dt} = (r_{ij} - l_{ij})\,\tfrac{1}{\tau}, \tag{S10}$$

where τ is the relaxation time. We model the dynamics of myosin activity (active stress intensity) in terms of mechanisms for myosin recruitment and tension-dependent dissociation

$$\frac{dm_{ij}}{dt} = \left(\tfrac{m_n}{t_r} - \tfrac{m_{ij}}{t_d} e^{-k_o T_{ij}} - \chi m_{ij}\right) \tfrac{t_p}{t_c}, \tag{S11}$$

where t_r is the recruitment time scale of myosin from the cytoplasm, which has a given maximum concentration m_n, and $t_d^{-1} e^{-k_o T_{ij}}$ represents the tension-dependent dissociation rate of actomyosin cables. χ has units $[t^{-1}]$ and accounts for the saturation of myosin activity on junctions, ensuring that active stress cannot accumulate without bound. Physically, available myosin may be depleted from basal pools or the number of active myosin motors that can operate on actin filaments may be limited. In this work, we remain agnostic as to which saturation mechanisms may operate and their relative contributions, so we capture saturation effects with a simple linear term. Later, we show that our results are not sensitive to the saturation mechanism. Note again that m, m_n have units $[Pa]$ as they describe the stress magnitude arising from active myosin. From the time when active myosin is available to the time it generates active stresses, there is a time scale (t_p). We model this delay with the ratio (t_p/t_c), where t_c is the characteristic time scale of the system.

Equations (S9-S11) model the coupled dynamics of tension and myosin activity in a single junction (Figure S1B). To couple the dynamics of adjacent edges and model a supracellular actomyosin cable, we further assume relaxational vertex dynamics. Restricting the cable to one dimension, the dynamics of each vertex are dominated by its two neighboring cable edges as

$$\frac{dr_i}{dt} = \frac{1}{\nu}\left(T_{i,i+1} - T_{i,i-1}\right), \tag{S12}$$

where ν represents effective friction resisting vertex displacement (*37, 35, 53*). The effective friction models the internal dissipative mechanisms such as the interaction of the cell junction with the cytosol and the viscous flow induced in the cytoskeleton due to the motion of the cell vertices (*28*). External dissipation by the surrounding fluid is assumed to be minimal. Thus, the length r_{ij} between vertices experiences a strain rate

$$\frac{dr_{ij}}{dt} = \frac{dr_j}{dt} - \frac{dr_i}{dt} = \frac{1}{\nu}\left(T_{i,j+1} - T_{i,j} - T_{i,j} + T_{i-1,i}\right) = \frac{\Delta T_{ij}}{\nu}, \tag{S13}$$

where Δ is the discrete Laplacian. In the continuum limit, we replace the discrete laplacian with $\Delta \bar{l}^2$, where $\bar{l}$ is the characteristic junction length (*37*). This provides two coupled equations for the dynamics of tension and myosin

activity:

$$\begin{cases} \frac{\partial T}{\partial t} = \frac{K\bar{l}^2}{\nu}\Delta T - \frac{T-m}{\tau} + \left(\frac{m_n}{t_r} - \frac{m}{t_d}e^{-k_o T} - \chi m\right)\frac{t_p}{t_c} \\ \frac{\partial m}{\partial t} = \left(\frac{m_n}{t_r} - \frac{m}{t_d}e^{-k_o T} - \chi m\right)\frac{t_p}{t_c}. \end{cases} \tag{S14}$$

These equations can be combined to give a differential equation for the difference between T and m

$$\frac{\partial(T-m)}{\partial t} = \frac{K\bar{l}^2}{\nu}\Delta T - \frac{T-m}{\tau}, \tag{S15}$$

which can also be understood as a differential equation for the elastic (or passive) component of the tension. The first term causes elastic tension to propagate along cables. The second term causes elastic tension to diminish as the rest length relaxes. Where the propagation term dominates, we expect transient deviations between m and T. Otherwise, the dynamics tend to reach $T = m$. Simulating equations (S14) with an initial central peak of active stress intensity, we find that, when $\frac{K\bar{l}^2\tau}{\nu}$ is small, T and m follow one another closely, as shown in Movie S13 and Figure S1C.

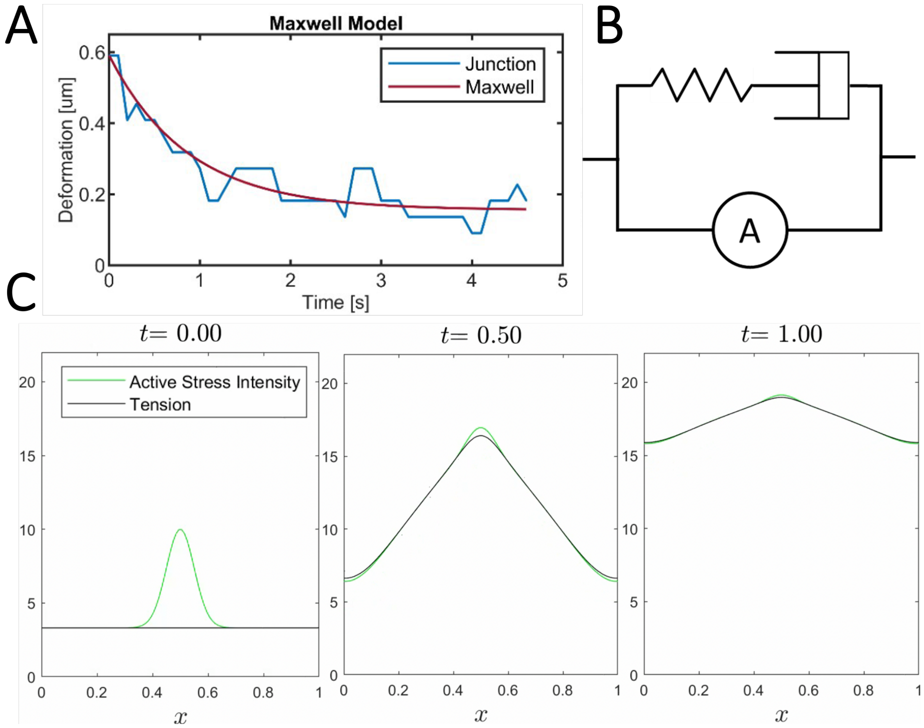

Supplementary Figure S1: **Mechanical feedback along a 1D cable.** A) Chick embryo experimental data from (*54*) shows that a junction deformed through optical manipulation exhibits a response that is best fit by Maxwellian viscoelasticity. B) Mechanical element for an actomyosin cable, consisting of a Maxwell element in parallel with an active element. C) Plots from the evolution of (S14) for sample parameters $\frac{K\bar{l}^2}{\nu} = 1$, $\tau = 0.001$, and $\chi\frac{t_p}{t_c} = 2.5$ so that cable tension reaches a maximum of $m = T = 20$. Sample parameters: $\frac{m_n t_p}{t_r t_c} = 50$, $\frac{t_p}{t_d t_c} = 100$, $k_o = 0.625$, $dx = 0.01$, $dt = 0.00001$.

1.3.2 Tissue-level modeling and time scales

The tissue-scale behavior is fluid-like and hence well described by viscous and active stresses (eq.(S5)) at gastrulation time scales. Extending the above reasoning to the tissue scale, active myosin contributes to cable tension T_c through an active stress contribution $T_A = \langle \mathbf{e}, \sigma_A \mathbf{e}\rangle = \frac{m}{2}$. At the junction level, T_A corresponds to m_{ij}, leaving $K(r_{ij} - l_{ij})$ to be accounted for. One can also compute from the viscous stress $T_V = \langle \mathbf{e}, \sigma_V \mathbf{e}\rangle$. However, σ_V and T_V reflect

fluid-like tissue stresses arising from cell division and intercalation. If and how T_V may affect junction-level cable tension is unclear. Regardless, we expect this effect to be of secondary importance because myosin-induced (active) tension (T_A) dominates T_V (*37*).

We are not aware of estimates for K or ν, so we cannot precisely estimate $\frac{K\bar{l}^2}{\nu}$. Moreover, the junction-level modeling presumes propagation over a 1D cable with no constraints on its extent. In the tissue, however, super-cellular cables have a limited extent spanning from 2 to 8 cells (*5*). Therefore, even if K and ν were known, propagation strength should be reduced by the effective length of super-cellular cables, which is small compared to the tissue scale. Last, the junction relaxation time has been estimated to be in the order of seconds in the chick epiblast (*54*). Altogether, the fast relaxation of the junction rest length and a small $\frac{K\bar{l}^2}{\nu}$ suggests that the elastic component of the tension is negligible at leading order. Following brief periods of rest length relaxation, mechanical feedback adjusts myosin activity to balance the tension of neighboring junctions (*35*). This is consistent with experimental observations that levels of myosin activity can serve as a readout for tension on cell junctions (*2*). Thus, in our coarse-grained continuum model, which neglects short-timescale elasticity and considers longer timescales over which the epithelium is effectively viscous, we expect

$$T_c \approx T_A = \frac{m}{2}, \tag{S16}$$

and that m will exhibit some effective propagation of active stress intensity along cables in their predominant orientation ϕ. In two dimensions, the effects of this directional propagation can be captured by two directional derivatives along the cable, $\Delta_\phi = D_\phi D_\phi$, and with a coefficient ξ modulating the tissue-scale effect of tension propagation. The definition $\Delta_\phi = D_\phi D_\phi$ is a simplification of the more general $(\mathbf{e} \cdot \nabla)^2$ and assumes that the individual cables along which propagation occurs are approximately straight locally.

We reason that at the tissue scale, the dynamics of active stress intensity are governed by similar mechanisms of myosin recruitment and tension-dependent dissociation modeled at the junction scale. Using the above assumptions and eq. (S16), we model the evolution of active stress magnitude as

$$m_t = \left(\frac{m_n}{t_r} - e^{-k_o \frac{m}{2}} \frac{m}{t_d} - \chi m\right)\frac{t_p}{t_c} - [\mathbf{v} \cdot \nabla]m + \xi \Delta_\phi m. \tag{S17}$$

The last two terms account for the advection of active stress intensity through material transport of actomyosin cables and for the directed propagation of active stress intensity along actomyosin cables with Δ_ϕ defined as the second directional derivative along the cable direction ϕ.

1.4 Model in Cartesian coordinates

The full model describing our active continuum can be summarized as

$$\begin{cases} \mathbf{0} = & u_{xx}\frac{1+2\mu c}{2c} + u_{yy}\mu + \frac{v_{xy}}{2c} + \frac{p_0 m_x}{2} + \frac{1}{2}(\cos 2\phi\ (2m\phi_y + m_x) + \sin 2\phi(m_y - 2m\phi_x)) \\ & v_{xx}\mu + v_{yy}\frac{1+2\mu c}{2c} + \frac{u_{xy}}{2c} + \frac{p_0 m_y}{2} + \frac{1}{2}(\sin 2\phi\ (2m\phi_y + m_x) - \cos 2\phi(m_y - 2m\phi_x)) \\ \phi_t = & \lambda(\frac{u_y+v_x}{2}\cos 2\phi + \frac{v_y-u_x}{2}\sin 2\phi) - [\mathbf{v}\cdot\nabla] + \frac{\omega}{2} \\ m_t = & (\frac{m_n}{t_r} - e^{-k_o\frac{m}{2}}\frac{m}{t_d} - \chi m)\frac{t_p}{t_c} - [\mathbf{v}\cdot\nabla]m + \xi\Delta_\phi m \\ & + \text{Boundary Conditions and Initial Conditions.} \end{cases} \tag{S18}$$

1.4.1 Non-dimensional model in Cartesian coordinates

Denoting by $u_c, x_c, t_c = x_c/u_c$ and $m_c = \mu u_c/x_c$ the characteristic velocity, characteristic length scale, characteristic time scale and the characteristic viscous shear stress, we rewrite eq. (S18) in non-dimensional compact form as

$$\begin{cases} \mathbf{0} = & 2p_1\Delta\mathbf{v} + \nabla[\nabla\cdot\mathbf{v}] + 2p_1\mathbf{B}\nabla m + \ p_1(p_0-1)\nabla m + 2p_1\nabla\cdot\mathbf{B}m \\ \phi_t = & \frac{p_2}{2}[\sin 2\phi(v_y-u_x) + \cos 2\phi(u_y+v_x)] - [\mathbf{v}\cdot\nabla]\phi + \frac{\omega}{2} \\ m_t = & p_3 - p_4 e^{-\frac{p_5}{2}m} m - p_6 m - [\mathbf{v}\cdot\nabla]m + p_7\Delta_\phi m \\ & + \text{Boundary Conditions and Initial Conditions,} \end{cases} \tag{S19}$$

where

$$\mathbf{B} = \mathbf{e}\otimes\mathbf{e} = \begin{pmatrix} \cos^2\phi & \sin\phi\cos\phi \\ \sin\phi\cos\phi & \sin^2\phi \end{pmatrix},$$

and m, ϕ, u, v, x, y, t are nondimensional. In eq. (S19), there are seven nondimensional parameters

$$p_0, \quad p_1 = \mu c, \quad p_2 = \lambda, \quad p_3 = \frac{t_p}{t_r}\frac{m_n}{m_c}, \quad p_4 = \frac{t_p}{t_d}, \quad p_5 = \frac{k_o\mu u_c}{x_c}, \quad p_6 = \chi t_p, \quad p_7 = \frac{\xi}{x_c u_c}. \tag{S20}$$

p_0 describes the cell's propensity of ingressing from a myosin-induced apical isotropic contraction and can be thought of also as the ratio of isotropic to anisotropic active stresses. p_1 describes the ratio of the shear to bulk viscosity, p_2 is the alignment parameter that describes how the actomyosin cables tend to orient due to shear-induced rotations and is ≈ 1 for elongated fibers. p_3 represents the ratio of the myosin (or active stress intensity) recruitment time scale t_r, from the ambient myosin pool of concentration m_n/m_c, to the characteristic t_p time scale, multiplied by m_n/m_c. p_4 represents the ratio of the t_p time scale to the dissociation time scale t_d. p_5 is the product of the characteristic viscous shear stress and k_o, which represents the inverse of the characteristic tension of the actomyosin cables (with units $[Pa]^{-1}$). p_6 is the strength of the simplest linear saturation term set by the ratio of t_p to the characteristic dissociation time scale χ^{-1} controlling saturation. Practically, p_6 enables setting a desired saturation level for m. p_7 is the ratio between transport of the active stress magnitude via tension propagation (with effective diffusion constant ξ) and active stress magnitude transport via advection.

The first two terms of the force balance are a viscous force and a force arising from compressibility inhomogeneities. Instead, the last three terms are active forces induced by m and ϕ inhomogeneities. Finally, observing the first two terms in the last equation of (S19), we note that more myosin induces higher tension on the cables, which in turn decreases the dissociation rate: $(m\uparrow) \rightarrow T_c\uparrow$ (more positive) $\rightarrow$ diss. rate $\downarrow\ \rightarrow$ myosin increases $(m\uparrow)$ (Figure 2A). This observation suggests an instability, which will become clear in section 1.4.3.

1.4.2 Role of p_0

To gain insights on p_0, we rewrite the force balance as

$$\nabla[\nabla\cdot\mathbf{v}] = p_1(1-p_0)\nabla m - 2p_1\mathbf{B}\nabla m - 2p_1\Delta\mathbf{v} - 2p_1 m\nabla\cdot\mathbf{B}. \tag{S21}$$

From eq. (S6), we know that $p_0 \geq 0$ reflects the expectation that high myosin induces isotropic apical cell contraction that causes active cell ingression. To elucidate the effect of p_0 on the relation between $\nabla[\nabla\cdot\mathbf{v}]$ and ∇m, we neglect the shear force $(-2p_1\Delta\mathbf{v})$, assume a simplified uniform ridge of active myosin in the y direction (Figure S2), and a spatially uniform cable distribution (i.e., $\nabla\cdot\mathbf{B} = \mathbf{0}$). Therefore, eq. (S21) simplifies to

$$\nabla[\nabla\cdot\mathbf{v}] \approx p_1(1-p_0)\nabla m - 2p_1\mathbf{B}\nabla m. \tag{S22}$$

If cables are perpendicular to the myosin ridge (Figure S2A), as in the wild-type development (Figure 1E at HH3 and Figure 2C right), eq. (S22) reveals that for all $p_0 \geq 0$, ∇m and $\nabla[\nabla\cdot\mathbf{v}]$ have opposite directions, reflecting the expectation that a ridge of high myosin induces a sink in the velocity field (Figure 2H). By contrast, if cables are

parallel to the myosin ridge (Figure S2B-C) as in the case of perturbation 3 (Figure 4 of (*4*) and Movie8), eq. (S22) reveals that for all $p_0 > 1$ (Figure S2B), ∇m and $\nabla[\nabla \cdot \mathbf{v}]$ have opposite directions. On the contrary, if $p_0 < 1$, ∇m and $\nabla[\nabla \cdot \mathbf{v}]$ would have the same direction (Figure S2C). Therefore, combining our model with experimental observations, we use $p_0 > 1$ when active cell ingression is present, and $p_0 = 0$ otherwise.

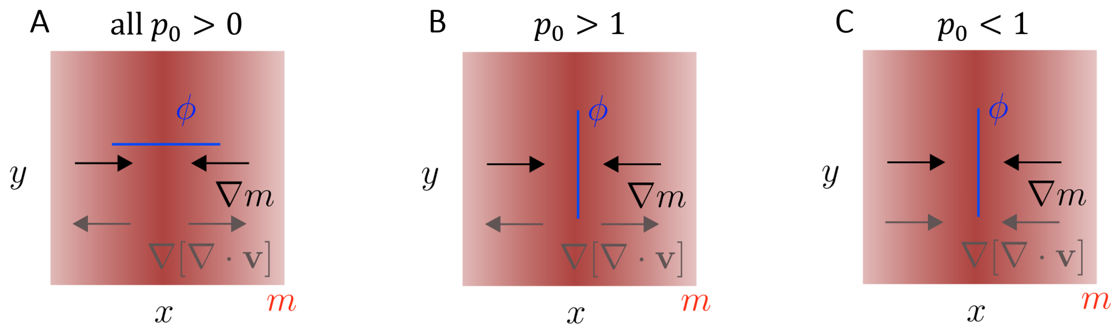

Supplementary Figure S2: **Influence of p_0 on the relationship between ∇m and $\nabla[\nabla \cdot \mathbf{v}]$.** We consider a simplified force balance (eq. (S22)) with no shear forces, a simple distribution m made of a ridge in the y direction and uniform actomyosin cables (i.e., $\nabla \cdot \mathbf{B} = \mathbf{0}$) perpendicular (A) or parallel (B, C) to the myosin ridge. Equation (S22) reveals that when cables are perpendicular (A) to the ridge, ∇m and $\nabla[\nabla \cdot \mathbf{v}]$ have opposite directions for all values of $p_0 > 0$, reflecting the expectation that a ridge of high myosin induces a sink in the velocity field. When cables are perpendicular to the ridge, ∇m and $\nabla[\nabla \cdot \mathbf{v}]$ have opposite directions for $p_0 > 1$ (B), and the same direction for $p_0 < 1$.

1.4.3 One-dimensional model

To gain insights about the model (S19), we assume slow dynamics in the $y-$direction, ignore the $\phi-$dynamics and set $\phi = 0$, obtaining the following simplified one-dimensional model

$$\begin{cases} 0 = u_{xx}(1+2p_1) + p_1\, m_x(1+p_0) \\ m_t = p_3 - p_4 e^{-\frac{p_5}{2}m} m - p_6 m - m_x u + p_7 m_{xx}. \end{cases} \tag{S23}$$

Equation (S23) describes the dynamics along a straight line perpendicular to the primitive streak. We set Dirichlet boundary conditions on the velocity $u(0,t) = -|u_b|$, $u(L,t) = |u_b|$ consistent with the symmetric cell migration in the extra-embryonic area, and no-flux boundary conditions for m: $m_x(0,t) = m_x(L,t) = 0$.

We investigate the main features of eq. (S23) by seeking the constant fixed points for m which solve the implicit equation $p_3 - (p_4 e^{-\frac{p_5}{2}m} + p_6)m = 0$. From the first equation, we compute the corresponding equilibrium velocity field $u_{eq.} = \frac{2|u_b|}{L}x - |u_b|$. We then study the linear stability of these equilibrium solutions by computing the spectrum of the corresponding linearized PDE. Denoting by $\delta\mathbf{h} = [\delta u, \delta m]^\top$ the perturbation from the equilibrium, we compute the linearized version of Eq. (S23) in operator form as $\mathcal{A}\delta\mathbf{h} = \mathcal{B}(x)\delta\mathbf{h}$ where

$$\mathcal{A} = \begin{bmatrix} 0 & 0 \\ 0 & \partial_t \end{bmatrix}, \; \mathcal{B}(x) = \begin{bmatrix} (1+2p_1)\partial_{xx} & p_1(1+p_0)\partial_x \\ 0 & \frac{1}{2}e^{-\frac{1}{2}p_5 m_{eq.}} p_4(p_5 m_{eq.} - 2) - (\frac{2|u_b|}{L}x - |u_b|)\partial_x - p_6 + p_7\partial_{xx} \end{bmatrix}, \tag{S24}$$

whose boundary conditions are inherited from the nonlinear problem, and correspond to zero Dirichlet boundary conditions for δu and zero flux boundary conditions for δm. We discretize the linearized PDE using a centered finite differencing scheme (*56*) and compute the generalized eigenvalue problem associated with eq. (S24).

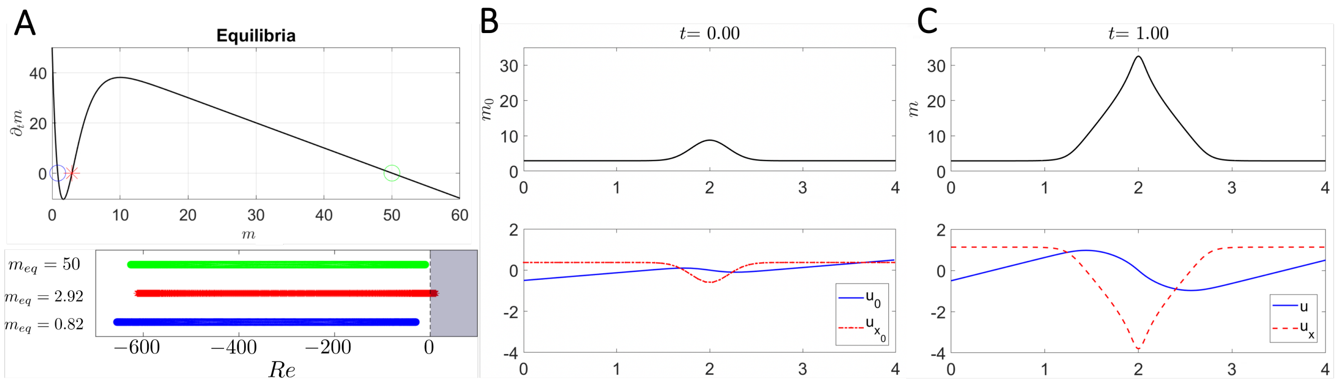

Supplementary Figure S3: **1D model**. (A) Top: Graph of $p_3 - (p_4 e^{-\frac{p_5}{2}m} + p_6)m = 0$ shows three $m_{eq.}$ marked with a blue circle, a red asterisk, and a green circle. Bottom: Truncated spectrum of the linearized PDE around corresponding $m_{eq.}$, $u_{eq.}$, consisting of the 500 eigenvalues with the highest real part. The spectrum at the intermediate equilibrium has positive real parts, implying that the intermediate equilibrium is linearly unstable. (B) The initial condition of the nonlinear 1D model using as initial myosin concentration a Gaussian added to the higher unstable $m_{eq.}$. The blue curve shows the associated initial velocity u_0, and the red dashed curve the initial velocity divergence u_{x_0}. (C) Same as B at the final time. The full time evolution is available as Movie S1. Sample parameters: $p_0 = 2$, $p_1 = 0.25$, $p_3 = 50$, $p_4 = 100$, $p_5 = 1.25$, $p_6 = 1$, $p_7 = 0.01$, $u_b = 0.5$, $L = 4$, $dx = 0.008$, $dt = 0.0001$.

Supplementary Figure S3A top shows three equilibria $m_{eq.}$, while the bottom panel shows the corresponding 500 eigenvalues, with the largest real part, of the linearized operator. The spectrum shows that the lowest and highest equilibria are linearly stable while the intermediate equilibrium is linearly unstable for the chosen parameter values. We confirm these linearized results by solving the full nonlinear PDE from an initial Gaussian m distribution (Figure S3B) added to the unstable $m_{eq.}$ (Figure S3A top, red asterisk). From a biological perspective, a region of higher m_0 represents the mesendoderm precursor. At later times (Figure S3C), m undergoes a focusing instability, that in turn generates a 1D attractor corresponding to the PS and marked by a peak of negative divergence (red dashed curve). The complete time evolution is available as Movie S1. Finally, the velocity divergence u_x shows a contracting region close to the PS as well as two symmetric expanding regions close to the boundary, consistent with the isotropic deformations observed in the Embryonic and Extra-Embryonic territories (*2*). Our simple 1D model reproduces several key features of amniote gastrulation. Yet, it is insufficient to reproduce the convergent extension and vortical patterns observed in experiments, which we investigate with our 2D model.

We note that eq. (S23) differs from the one-dimensional isotropic active gel instability in (*57*). Despite both associated with the dynamics of actomyosin on the cell cortex, eq. (S23) models myosin exchange with the cytoplasm as opposed to (*57*), which assume no exchange with the cytoplasm (eq. (1) and [8] in (*57*)). Additionally, our model accounts for catch-bonds between actin and myosin in the form experimentally measured in (*23*), which is absent in (*57*). Finally, (*57*) accounts for friction with the substrate, absent in our case. For a more detailed biological explanation behind the myosin exchange with the cytoplasm and the catch-bond mechanism in our context, we refer the reader to Figure 3 of (*5*).

1.5 Model in polar coordinates

Because of the circular geometry of the Extra-embryonic tissue and the symmetric radial cellular motion at its outer boundary, we write model (S18) in polar coordinates.

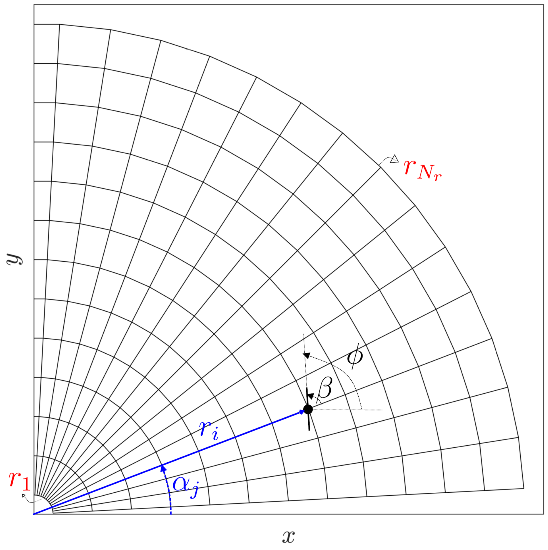

Supplementary Figure S4: **Numerical mesh.** Illustration of the discretization grid (first quadrant) used to solve eq. (S26). β denotes the cables orientation relative to α.

Denoting the velocity field by $\mathbf{v} = u(r,\alpha,t)\mathbf{e_r} + v(r,\alpha,t)\mathbf{e}_\alpha$ and the cables orientation relative to α by β (Figure S4), we obtain

$$\begin{cases} 0 = \frac{r^2(1+2c\mu)u_{rr}+2c\mu u_{\alpha\alpha}+r(1+2c\mu)u_r-(1+2c\mu)u-(1+4c\mu)v_\alpha+rv_{r\alpha}+p_0cr^2m_r+cr\sin 2\beta[m_\alpha-2rm\beta_r]+cr\cos 2\beta[rm_r+2m(1+\beta_\alpha)]}{2cr^2} \\ 0 = \frac{2c\mu r^2v_{rr}+(1+2c\mu)v_{\alpha\alpha}+2c\mu rv_r-2c\mu v+(1+4c\mu)u_\alpha+ru_{r\alpha}+p_0crm_\alpha+cr\sin 2\beta[2m(1+\beta_\alpha)+rm_r]-cr\cos 2\beta[m_\alpha-2rm\beta_r]}{2cr^2} \\ \beta_t = \frac{v-u_\alpha+rv_r}{2r} + \frac{\lambda}{2r}[\cos 2\beta(u_\alpha - v + rv_r) + \sin 2\beta(u + v_\alpha - ru_r)] - (\frac{v[1+\beta_\alpha]}{r} + u\beta_r) \\ m_t = (\frac{m_n}{t_r} - e^{-k_o\frac{m}{2}}\frac{m}{t_d} - \chi m)\frac{t_p}{t_c} - (\frac{vm_\alpha}{r} + um_r) + \xi[m_{rr}\cos^2\beta + m_{\alpha\alpha}\sin^2\beta + \left(\frac{m_{\alpha r}}{r} - \frac{m_\alpha}{2r^2}\right)\sin 2\beta] \\ u(r_{N_r},\alpha,t) = |u_b|,\ v(r_{N_r},\alpha,t) = 0,\ \beta_r(r_{N_r},\alpha,t) = 0,\ m_r(r_{N_r},\alpha,t) = 0 \\ + \text{Initial Conditions,} \end{cases} \tag{S25}$$

which, in non-dimensional form, becomes

$$\begin{cases} 0 = (u_{rr} - \frac{u}{r^2} + \frac{u_r}{r})(\frac{1+p_1}{p_1}) + \frac{u_{\alpha\alpha}}{r^2} + \frac{v_{r\alpha}}{rp_1} - \frac{v_\alpha}{r^2}(\frac{1+2p_1}{p_1}) + \frac{p_0m_r}{2} + \cos 2\beta(\frac{m[1+\beta_\alpha]}{r} + \frac{m_r}{2}) - \sin 2\beta(-\frac{m_\alpha}{2r} + m\beta_r) \\ 0 = v_{rr} + \frac{v_{\alpha\alpha}}{r^2}(\frac{1+p_1}{p_1}) + \frac{u_{r\alpha}}{rp_1} + \frac{v_r}{r} + \frac{u_\alpha}{r^2}(\frac{1+2p_1}{p_1}) - \frac{v}{r^2} + \frac{p_0m_\alpha}{2r} + \cos 2\beta(-\frac{m_\alpha}{2r} + m\beta_r) + \sin 2\beta(\frac{m[1+\beta_\alpha]}{r} + \frac{m_r}{2}) \\ \beta_t = \frac{v-u_\alpha}{2r} + \frac{v_r}{2} + \frac{p_2}{2}[\cos 2\beta(\frac{u_\alpha-v}{r} + v_r) + \sin 2\beta(-u_r + \frac{u+v_\alpha}{r})] - (\frac{v[1+\beta_\alpha]}{r} + u\beta_r) \\ m_t = p_3 - p_4e^{-\frac{p_5}{2}m}m - p_6m - (\frac{vm_\alpha}{r} + um_r) + p_7[m_{rr}\cos^2\beta + m_{\alpha\alpha}\sin^2\beta + \left(\frac{m_{\alpha r}}{r} - \frac{m_\alpha}{2r^2}\right)\sin 2\beta] \\ u(r_{N_r},\alpha,t) = |u_b|,\ v(r_{N_r},\alpha,t) = 0,\ \beta_r(r_{N_r},\alpha,t) = 0,\ m_r(r_{N_r},\alpha,t) = 0 \\ + \text{Initial Conditions.} \end{cases} \tag{S26}$$

2 Numerical Scheme

We develop a finite-difference numerical scheme to solve eq. (S26) in MATLAB. First, we multiply the momentum balance equations by r^2, leaving their solutions unaltered while avoiding numerical issues at grid points close to the origin. Then, we discretize spatial differential operators using centered finite-difference at second-order accuracy, except at the grid points (r_1, α_j) (Figure S4), where we use forward finite-difference at second-order accuracy (*56*).

We solve the first two equations for u, v by inverting (once) the corresponding discretized differential operator, which we multiply at each time step to the discretized forcing vector arising from the updated β, m-dependent terms. We adopt a two-step Adams–Bashforth method to solve the last two PDEs for β and α.

Because the cables orientation is a direction instead of a vector field, spatial derivatives of β need special care. Specifically, when computing $\beta_r(i,j) \approx \frac{\beta(i-1,j)-\beta(i+1,j)}{2\Delta r}$, one should make sure that both the sign and the amplitude are correct. We do so by rewriting $\frac{\beta(i-1,j)-\beta(i+1,j)}{2\Delta r} = \beta_r^p(i,j)+\beta_r^m(i,j)$, where $\beta_r^p(i,j) = \frac{\beta(i+1,j)-\beta(i,j)}{2\Delta r}$, $\beta_r^m(i,j) = \frac{\beta(i,j)-\beta(i-1,j)}{2\Delta r}$, and compute $\beta_r^p(i,j) = \text{sign}(\langle \mathbf{e}_{\beta(i+1,j)} \times \mathbf{e}_{\beta(i,j)}, \hat{\mathbf{e}}_z \rangle) \arccos \langle \mathbf{e}_{\beta(i+1,j)}, \mathbf{e}_{\beta(i,j)} \rangle$, where $\mathbf{e}_\beta = [\cos\beta, \sin\beta, 0]$, $\hat{\mathbf{e}}_z = [0,0,1]$. The same strategy can be used for higher-order derivatives and forward finite difference schemes. We summarize our numerical scheme in the following algorithm.

Algorithm 1 Numerical Solver of eq. (S26)

Inputs:
Grid: $r_i = i\Delta r,\ i = 1...N_r,\ N_r\Delta r = L, \alpha_j = \frac{\Delta\alpha}{2} + j\Delta\alpha,\ j = 0...N_{\alpha-1},\ N_\alpha\Delta\alpha = 2\pi$
Initial Conditions: $m(r,\alpha,0),\ \beta(r,\alpha,0)$
Parameters: $p_i,\ i = 0,..,7$

1. Solve for $u(r,\alpha,0),\ v(r,\alpha,0)$ from the first two equations
2. Compute $m(r,\alpha,\Delta t),\ \beta(r,\alpha,\Delta t)$ using $m(r,\alpha,0),\ \beta(r,\alpha,0),\ u(r,\alpha,0),\ v(r,\alpha,0)$
3. Repeat step 1 and 2 for all $t_k = k\Delta t,\ k = 0,..,N_t,\ T_f = N_t\Delta t$

Outputs: u, v, β, m at all grid points $\{r_i, \alpha_j, t_k\}$.

To prevent the development of sharp derivatives in β at the origin due to the forward finite-difference scheme, we add a diffusion term in the β_t equation with a non-dimensional prefactor of 10^{-3}. In all our simulations, we use $\Delta r = 0.05,\ L = 1,\ \Delta\alpha = 4^\circ, \Delta t = 5\times10^{-6},\ T_f \approx 1$. We monitor the accuracy of our numerical solver by substituting our solution at each time step into the momentum balance equations. We find deviations from zero of the order $\approx 10^{-11}$.

3 Induction of active stress intensity via tension propagation

To visualize the effect of directed propagation (Δ_ϕ) of active tension in polar coordinates, we solve the m_t equation in (S26) activating only the diffusion term on the right-end side. Figure S5A shows the evolution of active stress intensity m starting with high values in the sickle-shaped region of mesendoderm precursors. At later times, active stress intensity propagates around the epiblast due to the tension-propagation mechanism captured by $p_7\Delta_\phi m$ (Figure S5B). In the full model, however, all terms in eq. (S17) contribute to the evolution of m. Because the tissue is fluidized, the propagation of active stress intensity is dominated by advection so that active stress intensity does not appear to spread to the full extent depicted in Figure S5.

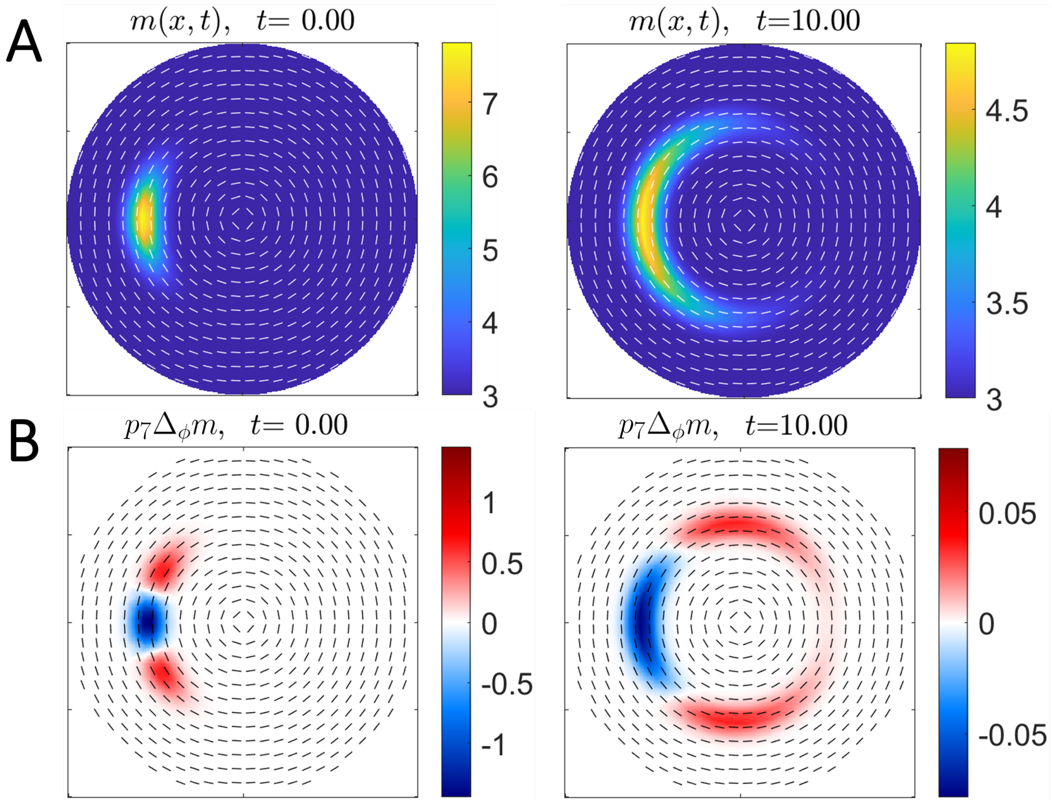

Supplementary Figure S5: **Visualization of directional propagation of active stress intensity.** A) Initial and final distribution of active stress intensity m under evolution $m_t = p_7\Delta_\phi m$ with no evolution of **v** or ϕ. B) Initial and final distribution of $p_7\Delta_\phi m$. Active stress orientations are indicated by white (A) and black (B) line segments. Sample parameter: $p_7 = 0.01$.

4 Parameters, boundary and initial conditions: selection and sensitivity analysis

In all our simulations, we use a single set of parameter values summarized in table 1.

p_0	p_1	p_2	p_3	p_4	p_5	p_6	p_7
0 or 2	0.15	0.9	50	100	1.25	1	0.01

Table 1: Parameter values.

We have selected parameter values using a combination of experiments, mechanistic arguments and numerical simulations; and have performed sensitivity analyses with respect to parameter changes as described below.

4.1 p_0, p_1, p_2

According to our arguments in Section 1.4.2, we set $p_0 = 0$ when active cell ingression is inhibited with drugs and $p_0 = 2$ otherwise. We use $p_1 = 0.15$ and note that increasing p_1 models a more compressible tissue. With higher p_1, the typical counter-rotating vortices (polonaise movement) disappear due to increased cell ingression and less cell redistribution (see Fig.S11). From Jeffery's equation (S8), p_2 modulates the rotation rate due to shear of a material fiber, which is ≈ 1 for elongated fibers. Here we set $p_2 = 0.9$, and note that slight variations of this value does not affect our results, as shown in WT simulations Movie S14 (Movie S15) for $p_2 = 0.6$ ($p_2 = 1$).

4.2 p_3, p_4, p_5

t_p is the approximate time required to observe active stress at the tissue scale generated by active myosin cables. This phenomenon involves a complex cascade of operations from kinetic reactions to single-cell and multicellular mechanochemical processes. Here we estimate t_p using the typical time for a junction to fully contract and induce an intercalation event driven by junctional myosin, a robust and experimentally accessible readout of tissue-scale active stress induced by actomyosin cables. Experiments in the chick epiblast show that this process takes up to $\approx 20min$ (*2*). A typical time course is shown in fig 4F of (*2*) where it takes 60 minutes for three junctions to successively contract. See also supplementary videos 10 and 11 of (*2*) which show various examples of intercalating cells, where it takes 4-10 frames (1 frame is 3 minutes) for junctions to contract. This result is consistent with tissue-scale mechanochemical models, where the actomyosin or active stress dynamics is the slowest (*37, 35, 58*), and a given configuration of active stress generates the corresponding tissue-scale velocity field by solving a static force balance (eq. 1 in S19). The recruitment and dissociation time scales are $t_r \simeq t_d \approx$ 10s in line with observations made in Drosophila (*53*). With these estimates, we set $p_3 = 50$ and $p_4 = 100$. p_5 is the coupling strength regulating myosin intensity and tension via the catch-bond feedback mechanisms. We are unaware of techniques to estimate p_5, and set $p_5 = 1.25$. To test the sensitivity of our results with respect to p_5, we show that a 20% reduction of the sensitivity to actomyosin dissociation rate to tension, induces a higher unstable myosin equilibrium concentration $m_{eq.}$ (Figure S6). The overall dynamic evolution of the system, however, remains qualitatively unchanged as shown in Movie S16, to be compared with Figure 2 and Movie S2.

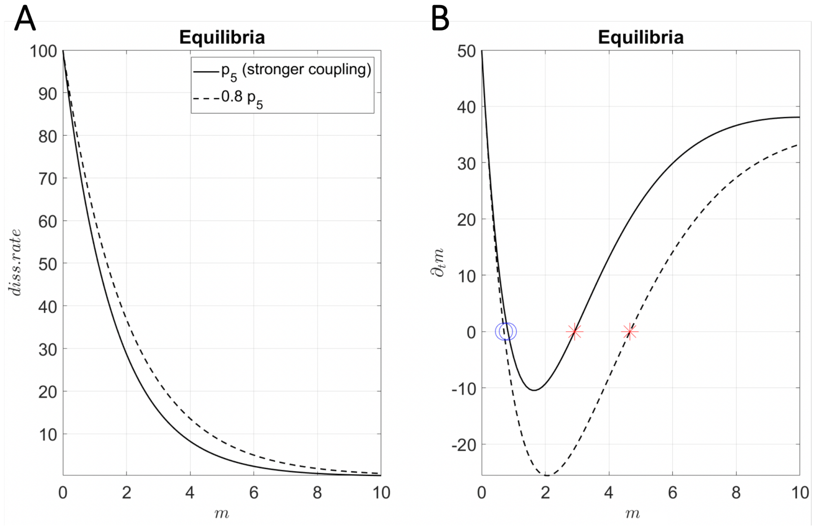

Supplementary Figure S6: **Sensitivity to p_5.** A) Exponential actomyosin dissociation rate $p_4 e^{-\frac{p_5}{2}m}$ for two values of p_5. B) Reducing p_5 by 20%, almost doubles the unstable m_{eq}.

By contrast, a 25% increase of p_5 causes a bifurcation of $m_{eq.}$, in which case model evolution differs considerably from experiments, as shown in Movie S17. Similar conclusions hold if changes in p_3 and p_4 induce a bifurcation of $m_{eq.}$. Our results, therefore, show that the active stress instability modulated by the catch-bond dynamics of actomyosin cables is essential for reproducing observed flow patterns.

4.3 p_6, p_7

While myosin activity cannot increase without bounds, the precise mechanisms by which and degree to which it saturates on junctions are beyond the scope of our model. This is because we are interested in a finite-time developmental interval characterized by instability rather than an asymptotic equilibrium state. To this end, we add a linear saturation

term $-p_6 m$ as the simplest functional form to dampen positive feedback and create a higher stable equilibrium value for m (Figure S3A and Figure S7A). For our model parameters, the three $m_{eq.}$ persist for $p_6 < 5.989$, preserving the instability needed for active stress intensity to grow. To show the robustness of our results with respect to changes in p_6, we show that even without ($p_6 = 0$) any saturation term (Figure S7B), the qualitative results of the 2D model do not change (Movie S18).

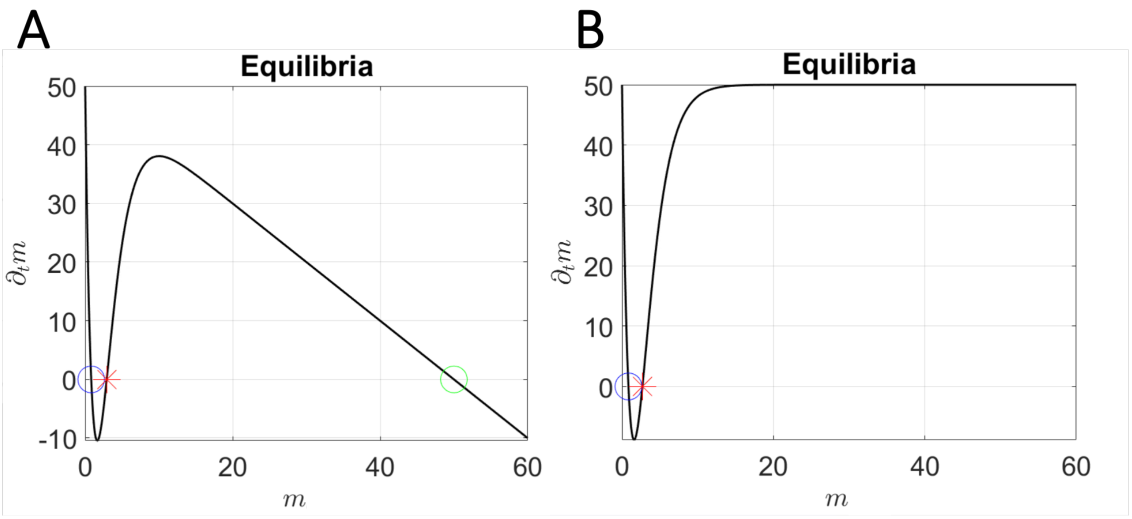

Supplementary Figure S7: **Sensitivity to p_6, controlling active stress intensity saturation.** Graphs of $p_3 - (p_4 e^{-\frac{p_5}{2} m} + p_6) m = 0$ for saturation parameter $p_6 = 1$ (A) and $p_6 = 0$ (B). Equilibria are marked with a blue circle (stable), red asterisk (unstable) and green circle (stable).

More generally, we find that slight variations in p_3, p_4, p_5, p_6 do not alter the overall system behavior as long as the graph (Figure S3A) determining m_{eq} does not change considerably (or undergoes a bifurcation).

Because the tissue is fluid-like and cells undergo embryo-scale motion, we argue that the advective transport of cells with active myosin dominates the induction of myosin via tension propagation on individual cables. Hence, we set $p_7 = 0.01$ throughout. Increasing the strength of active force propagation by 500% ($p_7 = 0.05$), the qualitative results of the 2D model (Movie S19) do not change, confirming the robustness of our results to parameters selection.

4.4 Boundary conditions

At the EP-EE boundary, the velocity is tangential to the boundary, ensuring that the EP area remains approximately constant over time, while in the EE region, the velocity is radial and roughly symmetric in α, explaining the expansion of the EE region over time (*2*). Because here we want to mechanistically predict what generates the observed flow patterns rather than enforcing it as an input, we do not select the boundary of our domain at the EP-EE interface, where the Dirichlet velocity boundary conditions would consist of a highly specific tangential flow profile. By contrast, we set our domain boundary $\partial\Omega$ (Figure 1A) slightly outside the EP-EE boundary, where, from the previous argument, the velocity is only radial and slightly higher than zero (we set $|\mathbf{v}_\mathrm{b}| = 0.2$). Remarkably, our model recapitulates the correct vortical flows within the EP region and the associated tangential velocity behavior at the EP-EE interface. The most natural choice of boundary conditions for m, ϕ is no flux, as there is no experimental evidence of sources, sinks, or constant values of m, ϕ close to the EP-EE boundary. Mathematically, we set $\mathbf{v} = \mathbf{v}_\mathrm{b}|_{\partial\Omega}$, $\nabla m \cdot \mathbf{n} = \mathbf{0}$, $\nabla \phi \cdot \mathbf{n} = \mathbf{0}$, where $\mathbf{n}$ is the outer normal to $\partial\Omega$.

To test the sensitivity of our results with respect to $|\mathbf{v}_\mathrm{b}|$, we find that a 50% increase (decrease) of $|\mathbf{v}_\mathrm{b}|$ does not affect the qualitative behavior of our results, as shown in Movie S20 (Movie S21) to be compared with Movie S2.

Overall, increasing (decreasing) $|\mathbf{v}_\mathrm{b}|$ exerts a stronger (weaker) pulling of the PS towards the boundary.

4.5 Initial conditions

Consistent with experiments (Figure 1E at HH1), we set the initial orientation of actomyosin cables $\phi(\mathbf{x}, t_0)$ along the tangential direction, and $m(\mathbf{x}, t_0)$ as a Gaussian distribution (Figure 2C left) added to the unstable $m_{eq.}$ equilibrium (Figure S3A), modeling the onset of high active myosin in the mesendoderm sickle territory. The Gaussian

$$m(\mathbf{x}, t_0) = m_{eq.} + Ae^{-\frac{(r-r_c)^2}{2\sigma_r^2} - \frac{(\alpha-\alpha_c)^2}{2\sigma_\alpha^2}} \quad \text{(reduced mesendoderm)} \tag{S27}$$

is centered at $r_c = 0.6, \alpha_c = 250°$, has amplitude $A = 5$ and standard deviations $\sigma_r = 0.06, \sigma_\alpha = 3\sigma_r/r_c$. We select $\alpha_c = 250°$ to align the AP axis of the model (Figure 2C) with the AP axis from the experiment (Figure 2E). Changing α_c only changes the AP axis orientation. We set $\sigma_\alpha = 3\sigma_r/r_c$ so that the Gaussian width in the tangential direction is three times the width in the radial direction, reflecting the asymmetry of the mesendoderm sickle.

To test the sensitivity of our results to our Gaussian perturbation, we find that a 20% increase (decrease) in A Movie S22 (Movie S23) does not affect the qualitative behavior of our model. Similar conclusions hold for a 20% increase (decrease) in σ_r Movie S24 (Movie S25) and for 20% increase (decrease) in r_c Movie S26 (Movie S27). Increasing r_c causes a stronger pulling of the PS towards the boundary.

4.6 Summary

In all our simulations of (S26) (i.e. eq. 1 in the main text) producing different gastrulation modes (Figures 2-5), we use a single set of parameter values and boundary conditions, summarized in table 2, except for the initial myosin distribution $m(\mathbf{x}, t_0)$ and the scalar, constant parameter p_0, which controls the ratio of isotropic (ingression) to anisotropic (intercalation) active stresses, as described in detail in SI Sec. 1.4.2.

p_1	p_2	p_3	p_4	p_5	p_6	p_7	epiboly velocity $\lvert\mathbf{v}_\mathbf{b}\rvert$
0.15	0.9	50	100	1.25	1	0.01	0.2

Table 2: Parameter values used in all two-dimensional simulations.

We change only p_0 and $m(\mathbf{x}, t_0)$. We set $p_0 = 2$ when there is active cell ingression and $p_0 = 0$ otherwise. Additionally, in the case of reduced mesendoderm precursors, we set $m(\mathbf{x}, t_0)$ as described in eq. (S27). By contrast, in the case of circular mesendoderm precursors (or ancestral condition), we use

$$m(\mathbf{x}, t_0) = m_{eq.} + Ae^{-\frac{(r-r_c)^2}{2\sigma_r^2}} \quad \text{(circular mesendoderm)} \tag{S28}$$

which models a Gaussian function of r, symmetric in α, added to the unstable $m_{eq.}$ and with the same parameters used in the wild type (Section 4.5). We summarize the combination of p_0 and $m(\mathbf{x}, t_0)$ to produce different gastrulation modes in Table 3.

gastrulation mode	p_0	$m(\mathbf{x}, t_0)$
Chick	2	reduced mesendoderm, eq.(S27)
Reptile	0	reduced mesendoderm, eq.(S27)
Teolost Fish	2	circular mesendoderm, eq.(S28)
Amphibian	0	circular mesendoderm, eq.(S28)

Table 3: Summary of p_0 and $m(\mathbf{x}, t_0)$ used to generate different gastrulation modes.

5 Comparison with other models

There is an increasing theoretical effort to couple mechanical and chemical signals in multicellular processes (*31, 32, 33, 34, 35, 36, 37, 3, 46, 38*). Our model has two main contributions. i) The tension-dependent dynamics of actomyosin cables (eqs. (S14),(S17)). ii) The coupling of tissue compressibility to tissue stress and active myosin (eq.(S6)). Regarding i), using a different argument in the context of active vertex models with no cell intercalations, (*37*) found for the first time that myosin recruitment along active edges must depend on the internal strain rate of the edge (or myosin cables) to guarantee the mechanical stability of the active tension network.

5.1 Differences with the tensile-ring model from (*3, 46*)

An alternative modeling approach for amniotic gastrulation is proposed in (*3, 46*) suggesting that a tensile ring at the EP-EE margin is sufficient to reproduce observation. We highlight below the main difference with our approach.

- Tensile ring model from (*3*) consists of a viscous flow

$$\begin{cases} \alpha \mathbf{\Delta v} = \nabla p + \mathbf{F_a}, \\ \nabla \cdot \mathbf{v} = \gamma, \quad \text{fitted from data in space and time,} \\ \mathbf{F_a} \text{ acts on a ring located at EP-EE margin, and is fitted from data in space and time.} \end{cases} \tag{S29}$$

  Instead of fitting $\mathbf{F_a}$ over space and time, (*46*) propose a mechanistic model to evolve $\mathbf{F_a}$ at later times starting from an observed initial distribution.

  By contrast, we model the fully coupled mechanochemical process and use information from experiments only at the initial time (eq. (S19)), hence providing a predictive mechanistic model. Specifically, we describe the process as a viscous flow

$$\begin{cases} \alpha \mathbf{\Delta v} = \nabla p + \mathbf{F_a}, \ \text{first two eqs. in (S19)}, \\ \nabla \cdot \mathbf{v} = \mathbf{g}(\mathbf{F_a}, \mathbf{v}, p), \ \text{eq.(S6)}, \\ \partial_t \mathbf{F_a} = \mathbf{f}(\mathbf{F_a}, \mathbf{v}), \ \text{second two eqs. in (S19)}. \end{cases} \tag{S30}$$

  Using only experimental actomyosin cables distribution at the initial time (HH1) and predicting experimental observations over the following twelve hours (up to HH3) is an important difference compared to the procedure proposed in (*3*) that requires data fitting over space and time.

- The active force $\mathbf{F_a}$ in (*3*) acts only on a ring centered at the EP-EE margin. By contrast, we model $\mathbf{F_a}$ in the whole embryo because actomyosin cables generating $\mathbf{F_a}$ are abundantly distributed and evolve dynamically within the embryo consistent with experimental observations (Figure 1). At stages after HH1, experiments show that active myosin is dominant along the PS rather than on the EP-EE boundary (Figures 1D-E), consistent with our model prediction (Figure 2C). To support further this finding, in Figure 3 we show $\mathbf{F_a}$ inferred from PIV velocity and eq. (S19) at HH3. The active forces are mainly perpendicular to the PS (Figures 3) rather than being organized on a ring-shaped structure.

- (*3, 46*) do not address the forces that drive γ (eq. (S29)), which is fitted from data. By contrast, our model (eq. (S30)) explains and predicts them in time as a response to mechanical stress, recapitulating the flow divergence and areal changes that we see in experiments (Figures 2F-H), as well as the overall Dynamic Morpphoskeletons and other Lagrangian metrics (Figure 2 and Movies 2-3).

Our ability to predict four different perturbations in addition to the wild-type development (Figs. 2,4 and Movies 2-12) supports the validity of our model and hence the underlying biological hypothesis.

6 The Dynamic Morphoskeletons quantify morphogenetic flows

To quantify spatio-temporal flows, we use the notion of Lagrangian coherent structures (*59*) adapted to morphogenesis, i.e. the the embryo's dynamic morphoskeletons (DM) (*44*). The DM is based on a Lagrangian description (Figure S8A) of tissue deformation captured by Finite Time Lyapunov Exponents (FTLE), which naturally combine local and global mechanisms along cell trajectories. The DM consists of attractors and repellers toward which cells converge or diverge over a specific time interval $T = t_f - t_0$. Repellers are marked by high values of the forward FTLE $\Lambda_{t_0}^{t_f}(\mathbf{x}_0)$; attractors are marked by high values of backward FTLE $\Lambda_{t_f}^{t_0}(\mathbf{x}_f)$ and their domain of attraction (DOA) by high values of the backward FTLE displayed on the initial cell positions $\Lambda_{t_f}^{t_0}(\mathbf{x}_0)$ (see Figure S8B for an illustration). The DM is objective, reveals the intrinsic geometric feature of spatio-temporal trajectories and is robust to noise (*44*), hence it is ideal for quantifying morphogenesis and comparing models with experiments.

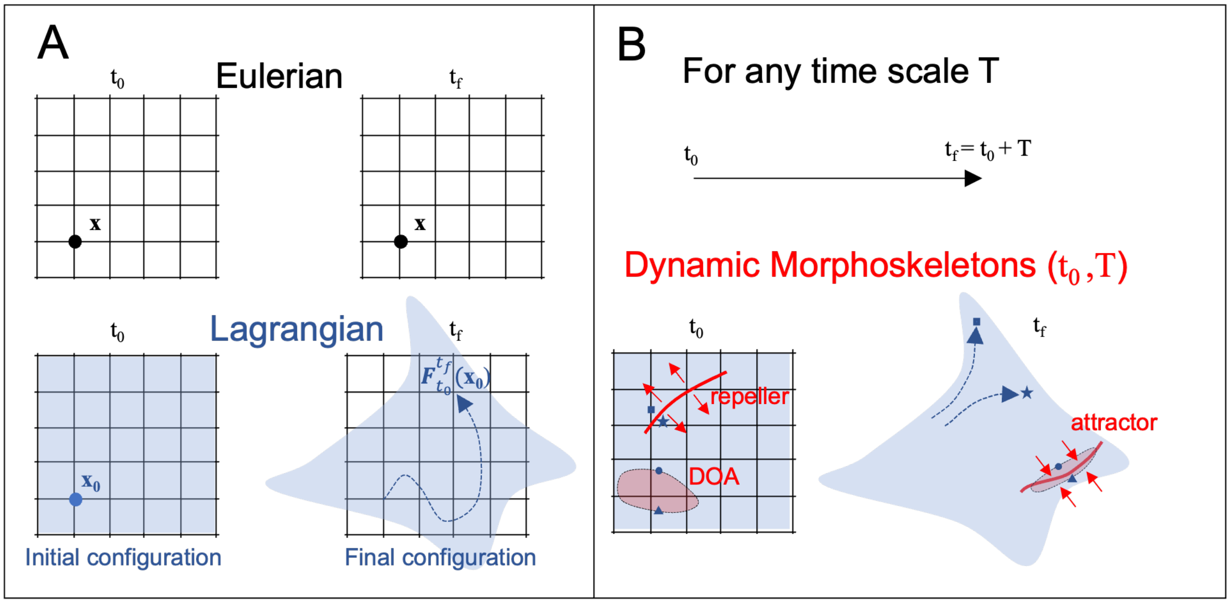

Supplementary Figure S8: **The Dynamic Morphoskeletons (*44*) quantify morphogenetic flows.** A) Eulerian coordinates $\mathbf{x}$ describe fixed spatial locations, while Lagrangian coordinates $\mathbf{x_0}$ label the identity of cells or tissue regions at their initial position and follow their trajectories $\mathbf{F}_{t_0}^{t_f}(x_0)$ over time. B) Fix the initial time t_0 of the Lagrangian analysis, which we consider to be the beginning of our experiments (developmental stage HH1). For any Lagrangian time scale T, the Dynamic Morphoskeletons consist of repellers, attractors and Domain of Attractions (DOA). Repellers mark regions at the initial cell configuration $\mathbf{x_0}$ such that nearby cells (star and square markers) at opposite sides of the repeller will separate by the final time t_f. Attractors mark regions at the final cell configuration $\mathbf{x_f}$ such that initially far cells (triangle and circle markers) will converge to the attractor by the final time t_f. The DOA (red area) marks the initial position of all the cells that will converge to the attractor by t_f.

Given a modelled or experimental planar velocity field $\mathbf{v}(\mathbf{x}, t)$, we compute the Dynamic Morphoskeleton (DM) (*44*) from the backward and forward Finite Time Lyapunov Exponents (FTLE). We compute the FTLE as

$$\Lambda_{t_0}^{t_f}(\mathbf{x}_0) = \frac{\ln(\lambda_2(\mathbf{x}_0))}{|t_f - t_0|}, \quad \mathbf{x}_f := \mathbf{F}_{t_0}^{t_f}(\mathbf{x}_0) = \mathbf{x}_0 + \int_{t_0}^{t_f} \mathbf{v}(\mathbf{F}_{t_0}^{\tau}(\mathbf{x}_0), \tau)\, d\tau, \tag{S31}$$

where $\lambda_2(\mathbf{x}_0)$ denotes the highest singular value of the Jacobian of the flow map $\nabla\mathbf{F}_{t_0}^{t_f}(\mathbf{x}_0)$ and $\mathbf{F}_{t_0}^{t_f}(\mathbf{x}_0)$ the flow map describing the trajectories from their initial $\mathbf{x}_0$ to final $\mathbf{x}_f$ positions. To compute the FTLE, we first calculate $\mathbf{F}_{t_0}^{t}(\mathbf{x}_0)$ by integrating the cell velocity field $\mathbf{v}(\mathbf{x}, t)$ using the MATLAB built-in Runge-Kutta solver ODE45 with absolute and relative tolerance of 10^{-6}, linear interpolation in space and time, and a uniform dense grid of initial conditions. Then,

denoting the $i-th$ component of the flow map $\mathbf{F}_{t_0}^t(\mathbf{x}_0)$ by $x^i(x_0^1, x_0^2, t_0, t)$, we compute the deformation gradient $\nabla\mathbf{F}_{t_0}^t(\mathbf{x}_0)$ using the finite-difference approximation

$$\nabla\mathbf{F}_{t_0}^t(\mathbf{x}_0) \approx \begin{bmatrix} \frac{x^1(x_0^1+\delta,x_0^2,t_0,t)-x^1(x_0^1-\delta,x_0^2,t_0,t)}{2\delta} & \frac{x^1(x_0^1,x_0^2+\delta,t_0,t)-x^1(x_0^1,x_0^2-\delta,t_0,t)}{2\delta} \\ \frac{x^2(x_0^1+\delta,x_0^2,t_0,t)-x^2(x_0^1-\delta,x_0^2,t_0,t)}{2\delta} & \frac{x^2(x_0^1,x_0^2+\delta,t_0,t)-x^2(x_0^1,x_0^2-\delta,t_0,t)}{2\delta} \end{bmatrix}, \tag{S32}$$

where δ is the initial conditions' grid spacing. After computing $\nabla\mathbf{F}_{t_0}^t(\mathbf{x}_0)$, we use eq. (S31) for computing the FTLE field.

7 Additional supplementary figures

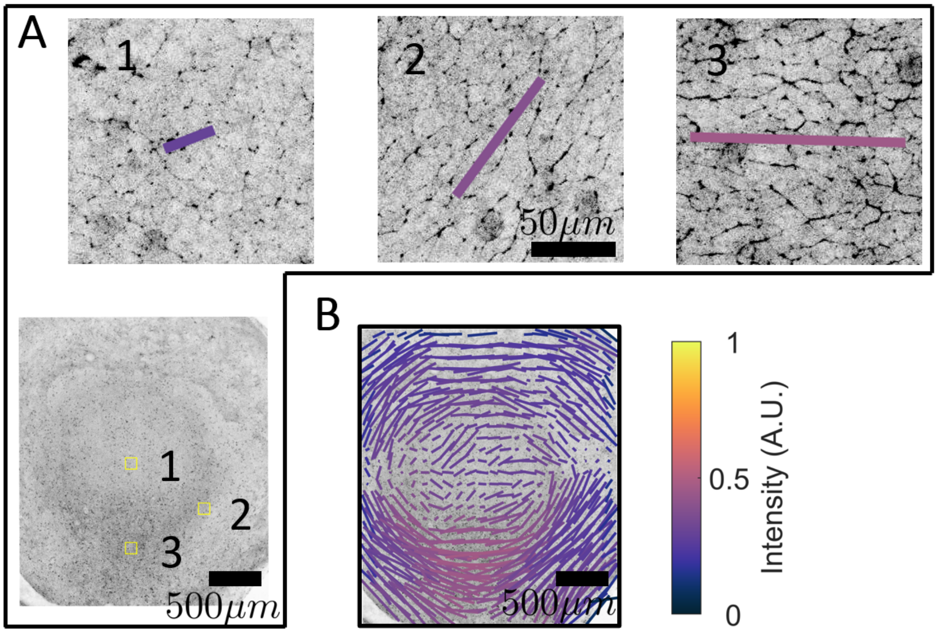

Supplementary Figure S9: **Actomyosin cables quantification.** A) Phospho-myosin light chain (pMLC) expression in the chick gastrula (HH1). Panels 1-3, taken at the positions of the yellow squares in the overview image, show the formation of pMLC cables spanning several cells. The length and orientation of the bars in panels 1-3 quantify the anisotropy and orientation of the pMLC cables, and the color of the bars indicates the pMLC concentration. B) The pattern of pMLC cables of the HH1 stage embryo shown in A. The alignment and the orientation of the active myosin cables are quantified from the asymmetry of the Fourier power spectrum (*11*), calculated in a tiling pattern over the embryo, while myosin intensity is calculated as average intensity in the same tiles. For experimental details, see the accompanying paper (*4*).

8 Additional model perturbations

In this section, we show the effects of additional model perturbations that provide insight into the role of p_1 and $\mathbf{m}(\mathbf{x}, t_0)$. All other parameters and initial and boundary conditions are the same as WT. First, by increasing the ratio of shear to bulk viscosity (p_1), the polonaise movements are lost (Figure S11A), resembling instead the impaired gastrulation movements experimentally observed in the absence of cell division (*60, 61*). We reason that, at leading order, impairing cell divisions reduces fluidity, which is captured by the increase of tissue-scale shear viscosity μ, and hence p_1 (*62*).

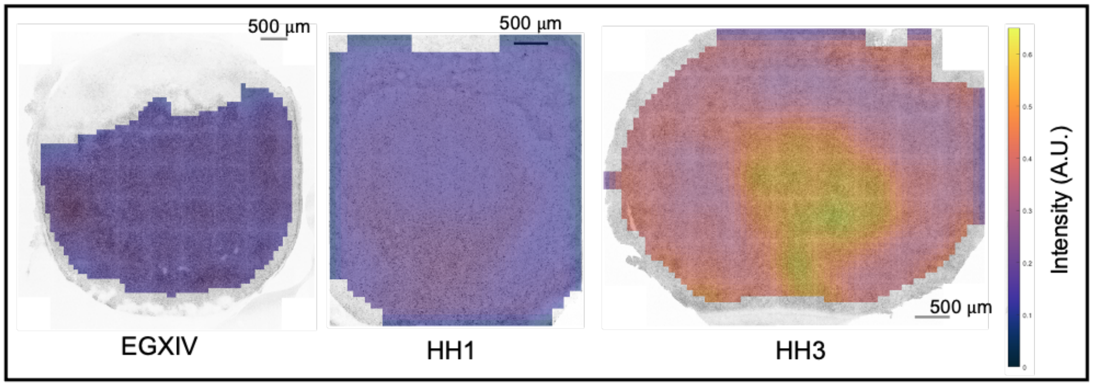

Supplementary Figure S10: **Quantification of active myosin via pMLC intensity.** The pattern of pMLC intensity evolves in time, as in Figure 1E. Color represents absolute measured signal intensity (arbitrary units). Changes in absolute pMLC intensity show a global increase in active myosin as gastrulation progresses, particularly from HH1 to HH3. This increase is consistent with the active stress instability predicted by our model (Figure 2). For experimental details, see the accompanying paper (*4*).

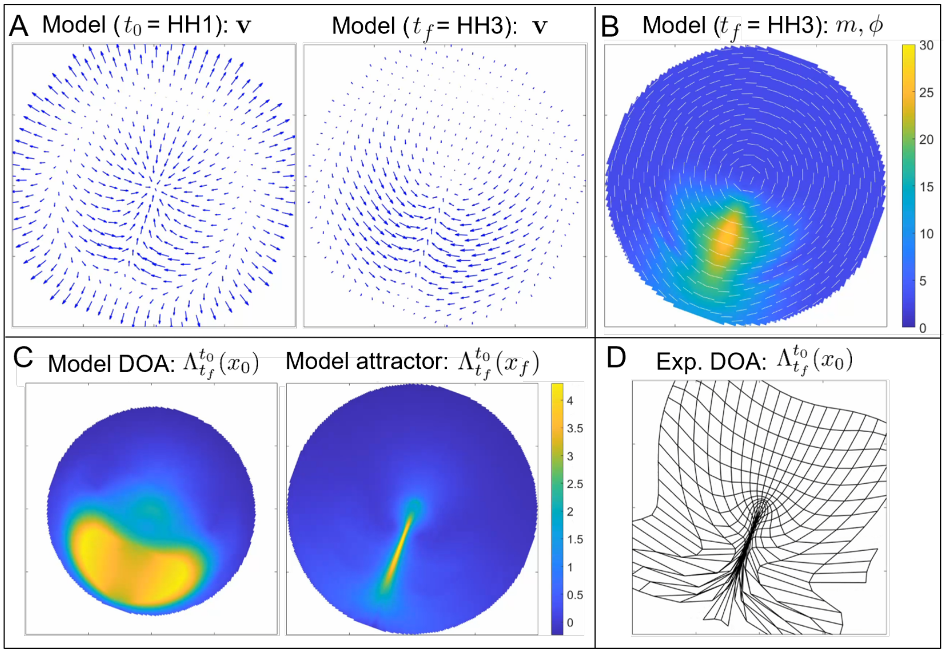

Supplementary Figure S11: **A higher ratio p_1 of shear and bulk viscosity destroys polonaise movements.** Model output with a higher $p_1 = 0.5$. A) Initial (t_0) and final (t_f) velocity field. B) Final (t_f) distribution of m, ϕ. C) Model-based DOA at t_0 (HH1) and the attractor at t_f (HH3). D) Deformed Lagrangian grid from the predicted model velocity. Units are non-dimensional.

Next, we vary the angular extent (σ_α) of the initial mesendoderm, encoded in $\mathbf{m}(\mathbf{x}, t_0)$. When σ_α is much smaller, a streak forms, but the attractor is shorter and its DOA is far smaller than WT (Figure S12A-D). By instead increasing σ_α, the reduced mesendoderm initial condition (eq. (S27)) can be gradually transformed into the circular mesendoderm initial condition (eq. (S28)). Figure S12E-H showcases the model predictions for an intermediate initial condition ($\sigma_\alpha = 0.8$). Here, active stress intensity is initialized in a ring with much greater stress in the posterior. While polonaise movements are generated and maintained, the DM (S12G) lacks a sharp attractor while having a large DOA caused by excessive contractile activity in the embryo, differing substantially from WT. These results suggest that the finite width of the mesendoderm precursor cells at HH1 (see experiments in Fig. 1E) is important

for generating the correct gastrulation flows. The finite extent of the mesendoderm precursor cells is generated by a molecular patterning of the embryo before HH1 (*5*).

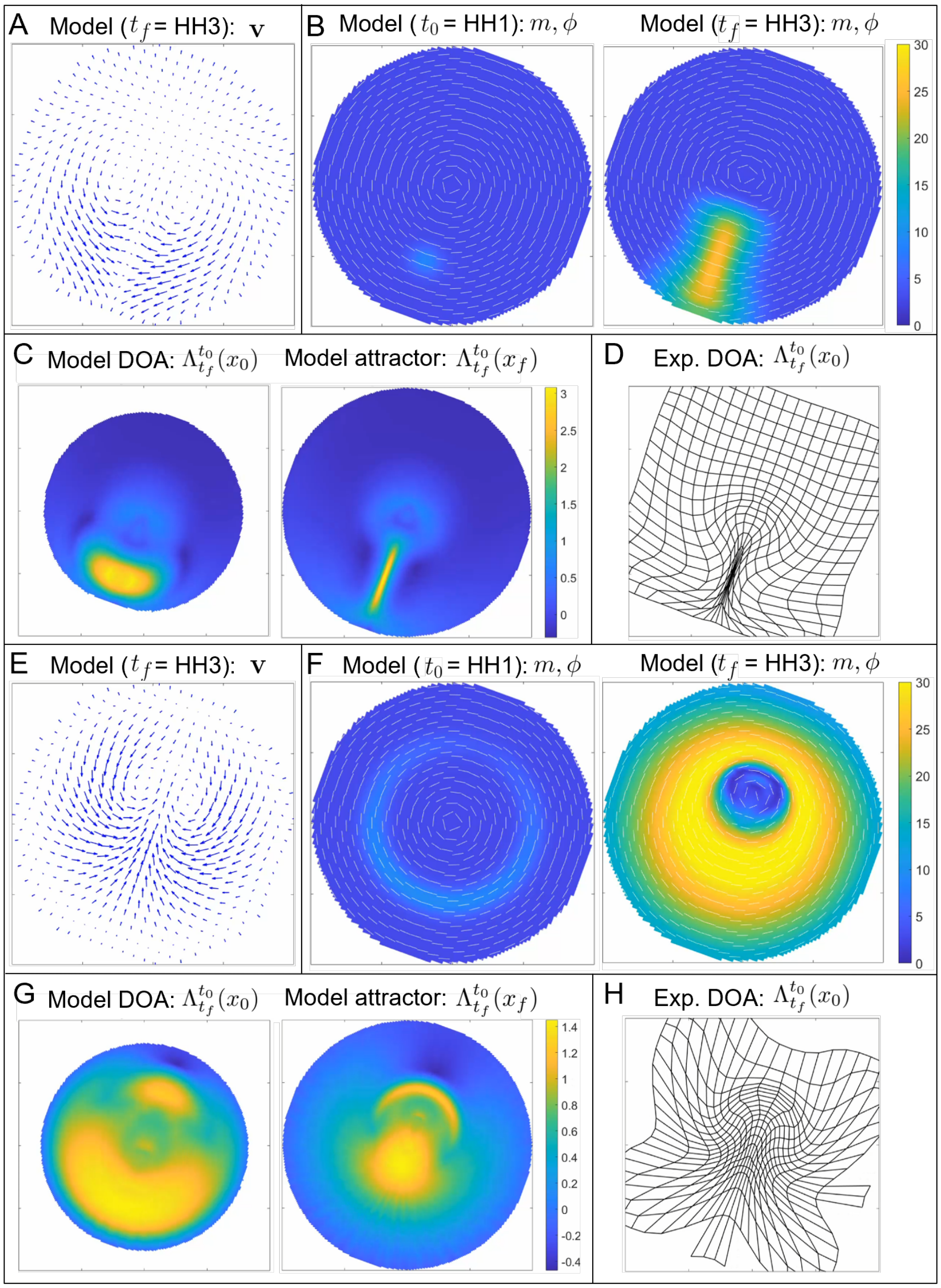

Supplementary Figure S12: **Effect of extent of initial mesendoderm.** Model output from initial conditions with a smaller mesendoderm $\sigma_\alpha = \sigma_r = 0.06$ (A-D) and an intermediate mesendoderm $\sigma_\alpha = 0.8$ (E-H) between the reduced mesendoderm and circular mesendoderm initial conditions. A,E) Final (t_f) velocity fields. B,F) Initial (t_0) and final (t_f) distributions of m, ϕ. C,G) Model-based DOA at t_0 (HH1) and the attractor at t_f (HH3). D,H) Deformed Lagrangian grids from the predicted model velocity. Units are non-dimensional.

Finally, we show instances in which two streaks form, either spontaneously or induced by implantation of Vg1 (GDF3) secreting cells (*63*). In this case, distinct streaks either maintain their separation or fuse. In the first case,

we initialize two reduced mesendoderms 180° apart. Unlike the ordinary twin perturbation, the model predicts that these two streaks will not fuse but will instead halt each other's extension from posterior to anterior and vice-versa S13A-D). In the second case, we nitialize two reduced mesendoderms 90° apart and reduce the amplitude to $A = 0.1$ for one of them. Despite the smaller reduced mesendoderm's weaker convergent extension, which is apparent in lower scalar values of its corresponding attractor (S13G), it causes the stronger streak to bend away from it, as in the ordinary twin perturbation.

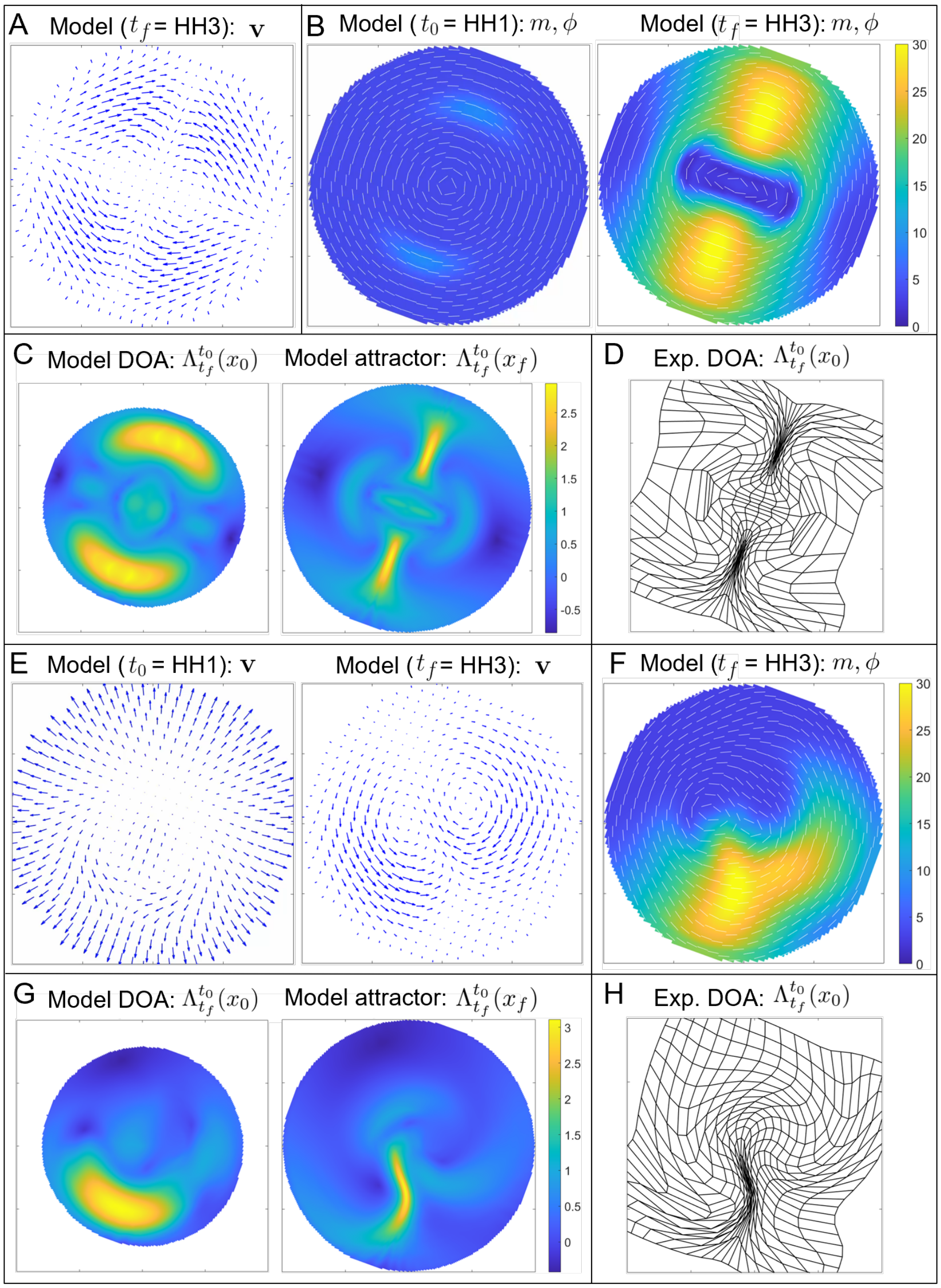

Supplementary Figure S13: **Multiple streaks.** Model output from initial conditions with two reduced mesendoderms oriented 180° apart (A-D) and 90° apart (E-H). A) Final (t_f) velocity field. B) Initial (t_0) and final (t_f) distributions of m, ϕ. C,G) Model-based DOA at t_0 (HH1) and the attractor at t_f (HH3). D,H) Deformed Lagrangian grids from the predicted model velocity. E) Initial (t_0) and final (t_f) velocity field. F) Final (t_f) distribution of m, ϕ. Units are non-dimensional.

Supplementary movies

Movie S1: Time evolution movie associated with Fig. 2B.
Movie S2: Time evolution movie associated with Figs. 2C-D.
Movie S3: Time evolution movie associated with Fig. 2E.
Movie S4: Time evolution movie associated with Fig. 4A.
Movie S5: Time evolution movie associated with Fig. 4B.
Movie S6: Time evolution movie associated with Fig. 4C.
Movie S7: Time evolution movie associated with Fig. 4D.
Movie S8: Time evolution movie associated with Fig. 4E.
Movie S9: Time evolution movie associated with Fig. 4F.
Movie S10: Deforming Lagrangian grid overlaid on the light-sheet microscope images corresponding to the experimental data used in Movie S9.
Movie S11: Time evolution movie associated with Fig. 4G.
Movie S12: Time evolution movie associated with Fig. 4H.
Movie S13: Time evolution movie associated with Fig. S1C.
Movie S14: Sensitivity to p_2. Same as Movie S2, but using a lower value of the parameter p_2.
Movie 15: Sensitivity to p_2. Same as Movie S2, but using a higher value of the parameter p_2.
Movie S16: Sensitivity to p_5. Same as Movie S2, but using a lower value of the parameter p_5.
Movie S17: Sensitivity to p_5. Same as Movie S2, but using a higher value of the parameter p_5.
Movie S18: Sensitivity to p_6. Same as Movie S2, but setting $p_6 = 0$.
Movie S19: Sensitivity to p_7. Same as Movie S2, but using a higher value of the parameter p_7.
Movie S20: Sensitivity to the epiboly velocity $|\mathbf{v_b}|$. Same as Movie S2, but using a higher value of $|\mathbf{v_b}|$.
Movie S21: Sensitivity to the epiboly velocity $|\mathbf{v_b}|$. Same as Movie S2, but using a lower value of $|\mathbf{v_b}|$.
Movie S22: Sensitivity to the amplitude A of the Gaussian perturbation added to the unstable m equilibrium. Same as Movie S2, but using a higher A.
Movie S23: Sensitivity to the amplitude A of the Gaussian perturbation added to the unstable m equilibrium. Same as Movie S2, but using a lower A.
Movie S24: Sensitivity to the radial standard deviation σ_r of the Gaussian perturbation added to the unstable m equilibrium. Same as Movie S2, but using a higher σ_r.
Movie S25: Sensitivity to the radial standard deviation σ_r of the Gaussian perturbation added to the unstable m equilibrium. Same as Movie S2, but using a lower σ_r.
Movie S26: Sensitivity to the radial mean r_c of the Gaussian perturbation added to the unstable m equilibrium. Same as Movie S2, but using a higher r_c.
Movie S27: Sensitivity to the radial mean r_c of the Gaussian perturbation added to the unstable m equilibrium. Same as Movie S2, but using a lower r_c.

REFERENCES AND NOTES

1. C. D. Stern, *Gastrulation: From Cells to Embryo* (CSHL Press, 2004).
2. E. Rozbicki, M. Chuai, A. I. Karjalainen, F. Song, H. M. Sang, R. Martin, H. J. Knölker, M. P. MacDonald, C. J. Weijer, Myosin-II-mediated cell shape changes and cell intercalation contribute to primitive streak formation. *Nat. Cell Biol.* **17**, 397–408 (2015).
3. M. Saadaoui, D. Rocancourt, J. Roussel, F. Corson, J. Gros, A tensile ring drives tissue flows to shape the gastrulating amniote embryo. *Science* **367**, 453–458 (2020).
4. M. Chuai, G. Serrano Nájera, M. Serra, L. Mahadevan, C. J. Weijer, Reconstruction of distinct vertebrate gastrulation modes via modulation of key cell behaviors in the chick embryo. *Sci. Adv.* **9**, eabn5429 (2023).
5. G. Serrano Nájera, C. J. Weijer, Cellular processes driving gastrulation in the avian embryo. *Mech. Dev.* **163**, 103624 (2020).
6. O. Voiculescu, F. Bertocchini, L. Wolpert, R. E. Keller, C. D. Stern, The amniote primitive streak is defined by epithelial cell intercalation before gastrulation. *Nature* **449**, 1049–1052 (2007).
7. Y. Nakaya, E. W. Sukowati, Y. Wu, G. Sheng, RhoA and microtubule dynamics control cell–basement membrane interaction in EMT during gastrulation. *Nat. Cell Biol.* **10**, 765–775 (2008).
8. O. Voiculescu, L. Bodenstein, I.-J. Lau, C. D. Stern, Local cell interactions and self-amplifying individual cell ingression drive amniote gastrulation. *eLife* **3**, e01817 (2014).
9. L. Gräper, Die Primitiventwicklung des Hühnchens nach stereokinematographischen Untersuchungen, kontrolliert durch vitale Farbmarkierung und verglichen mit der Entwicklung anderer Wirbeltiere. *Wilhelm Roux Arch. Entwickl. Mech. Org.* **116**, 382–429 (1929).
10. M. Chuai, W. Zeng, X. Yang, V. Boychenko, J. A. Glazier, C. J. Weijer, Cell movement during chick primitive streak formation. *Dev. Biol.* **296**, 137–149 (2006).
11. M. Durande, S. Tlili, T. Homan, B. Guirao, F. Graner, H. Delanoë-Ayari, Fast determination of coarse-grained cell anisotropy and size in epithelial tissue images using Fourier transform. *Phys. Rev. E* **99**, 062401 (2019).
12. C. Bertet, L. Sulak, T. Lecuit, Myosin-dependent junction remodelling controls planar cell intercalation and axis elongation. *Nature* **429**, 667–671 (2004).
13. J. T. Blankenship, S. T. Backovic, J. S. Sanny, O. Weitz, J. A. Zallen, Multicellular rosette formation links planar cell polarity to tissue morphogenesis. *Dev. Cell* **11**, 459–470 (2006).

14. J. A. Zallen, E. Wieschaus, Patterned gene expression directs bipolar planar polarity in drosophila. *Dev. Cell* **6**, 343–355 (2004).
15. J. Lavalou, Q. Mao, S. Harmansa, S. Kerridge, A. C. Lellouch, J.-M. Philippe, S. Audebert, L. Camoin, T. Lecuit, Formation of polarized contractile interfaces by self-organized Toll-8/Cirl GPCR asymmetry. *Dev. Cell* **56**, 1574–1588 (2021).
16. A. Munjal, J.-M. Philippe, E. Munro, T. Lecuit, A self-organized biomechanical network drives shape changes during tissue morphogenesis. *Nature* **524**, 351–355 (2015).
17. M. Rauzi, U. Krzic, T. E. Saunders, M. Krajnc, P. Ziherl, L. Hufnagel, M. Leptin, Embryo-scale tissue mechanics during Drosophila gastrulation movements. *Nat. Commun.* **6**, 8677 (2015).
18. S. J. Streichan, M. F. Lefebvre, N. Noll, E. F. Wieschaus, B. I. Shraiman, Global morphogenetic flow is accurately predicted by the spatial distribution of myosin motors. *eLife* **7**, e27454 (2018).
19. A. Bailles, C. Collinet, J. M. Philippe, P. F. Lenne, E. Munro, T. Lecuit, Genetic induction and mechanochemical propagation of a morphogenetic wave. *Nature* **572**, 467–473 (2019).
20. F. J. Vernerey, U. Akalp, Role of catch bonds in actomyosin mechanics and cell mechanosensitivity. *Phys. Rev. E* **94**, 012403 (2016).
21. R. Fernandez-Gonzalez, S. de Matos Simoes, J.-C. Röper, S. Eaton, J. A. Zallen, Myosin II dynamics are regulated by tension in intercalating cells. *Dev. Cell* **17**, 736–743 (2009).
22. J. M. Laakso, J. H. Lewis, H. Shuman, E. M. Ostap, Myosin I can act as a molecular force sensor. *Science* **321**, 133–136 (2008).
23. C. Veigel, J. E. Molloy, S. Schmitz, J. Kendrick-Jones, Load-dependent kinetics of force production by smooth muscle myosin measured with optical tweezers. *Nat. Cell Biol.* **5**, 980–986 (2003).
24. W. E. Thomas, V. Vogel, E. Sokurenko, Biophysics of catch bonds. *Annu. Rev. Biophys.* **37**, 399–416 (2008).
25. M. C. Marchetti, J. F. Joanny, S. Ramaswamy, T. B. Liverpool, J. Prost, M. Rao, R. A. Simha, Hydrodynamics of soft active matter. *Rev. Mod. Phys.* **85**, 1143–1189 (2013).
26. F. Graner, J. A. Glazier, Simulation of biological cell sorting using a two-dimensional extended Potts model. *Phys. Rev. Lett.* **69**, 2013–2016 (1992).
27. A. G. Fletcher, M. Osterfield, R. E. Baker, S. Y. Shvartsman, Vertex models of epithelial morphogenesis. *Biophys. J.* **106**, 2291–2304 (2014).
28. S. Alt, P. Ganguly, G. Salbreux, Vertex models: From cell mechanics to tissue morphogenesis. *Philos. Trans. R. Soc. Lond. B Biol. Sci.* **372**, 20150520 (2017).

29. A. Bonfanti, J. L. Kaplan, G. Charras, A. Kabla, Fractional viscoelastic models for power-law materials. *Soft Matter* **16**, 6002–6020 (2020).
30. R. Alert, X. Trepat, Physical models of collective cell migration. *Annu. Rev. Condens. Matter Phys.* **11**, 77–101 (2020).
31. C. Collinet, T. Lecuit, Programmed and self-organized flow of information during morphogenesis. *Nat. Rev. Mol. Cell Biol.* **22**, 245–265 (2021).
32. Y. Maroudas-Sacks, K. Keren, Mechanical patterning in animal morphogenesis. *Annu. Rev. Cell Dev. Biol.* **37**, 469–493 (2021).
33. B. Ladoux, R.-M. Mège, Mechanobiology of collective cell behaviours. *Nat. Rev. Mol. Cell Biol.* **18**, 743–757 (2017).
34. R. Etournay, M. Popović, M. Merkel, A. Nandi, C. Blasse, B. Aigouy, H. Brandl, G. Myers, G. Salbreux, F. Jülicher, S. Eaton, Interplay of cell dynamics and epithelial tension during morphogenesis of the Drosophila pupal wing. *eLife* **4**, e07090 (2015).
35. H. J. Gustafson, N. Claussen, S. De Renzis, S. J. Streichan, Patterned mechanical feedback establishes a global myosin gradient. *Nat. Commun.* **13**, 7050 (2022).
36. N. A. Dye, M. Popović, K. V. Iyer, J. F. Fuhrmann, R. Piscitello-Gómez, S. Eaton, F. Jülicher, Self-organized patterning of cell morphology via mechanosensitive feedback. *eLife* **10**, e57964 (2021).
37. N. Noll, M. Mani, I. Heemskerk, S. J. Streichan, B. I. Shraiman, Active tension network model suggests an exotic mechanical state realized in epithelial tissues. *Nat. Phys.* **13**, 1221–1226 (2017).
38. A.-C. Reymann, F. Staniscia, A. Erzberger, G. Salbreux, S. W. Grill, Cortical flow aligns actin filaments to form a furrow. *eLife* **5**, e17807 (2016).
39. E. W. Gehrels, B. Chakrabortty, M.-E. Perrin, M. Merkel, T. Lecuit, Curvature gradient drives polarized tissue flow in the *Drosophilaembryo*. *Proc. Natl. Acad. Sci. U.S.A.* **120**, e2214205120 (2023).
40. A. Ioratim-Uba, T. B. Liverpool, S. Henkes, Mechano-chemical active feedback generates convergence extension in epithelial tissue. arXiv:2303.02109 (2023). https://doi.org/10.48550/arXiv.2303.02109.
41. M. Valet, E. D. Siggia, A. H. Brivanlou, Mechanical regulation of early vertebrate embryogenesis. *Nat. Rev. Mol. Cell Biol.* **23**, 169–184 (2021).

42. G. Jeffery, The motion of ellipsoidal particles immersed in a viscous fluid. *Proc. R. Soc. Lond.* **102**, 161–179 (1922).
43. C. Truesdell, W. Noll, *The Non-Linear Field Theories of Mechanics* (Springer, 2004).
44. M. Serra, S. Streichan, M. Chuai, C. J. Weijer, L. Mahadevan, Dynamic morphoskeletons in development. *Proc. Natl. Acad. Sci. U. S. A.* **117**, 11444–11449 (2020).
45. G. Serrano Nájera, C. J. Weijer, The evolution of gastrulation morphologies. *Development* **150**, dev200885 (2023).
46. P. Caldarelli, A. Chamolly, O. Alegria-Prévot, J. Gros, F. Corson, Self-organized tissue mechanics underlie embryonic regulation. bioRxiv 2021.10.08.463661 (2021). https://doi.org/10.1101/2021.10.08.463661.
47. R. Feroze, J. H. Shawky, M. von Dassow, L. A. Davidson, Mechanics of blastopore closure during amphibian gastrulation. *Dev. Biol.* **398**, 57–67 (2015).
48. D. R. Shook, E. M. Kasprowicz, L. A. Davidson, R. Keller, Large, long range tensile forces drive convergence during Xenopus blastopore closure and body axis elongation. *eLife* **7**, e26944 (2018).
49. M. Valet, E. D. Siggia, A. H. Brivanlou, Mechanical regulation of early vertebrate embryogenesis. *Nat. Rev. Mol. Cell Biol.* **23**, 169–184 (2022).
50. A. Carmany-Rampey, A. F. Schier, Single-cell internalization during zebrafish gastrulation. *Curr. Biol.* **11**, 1261–1265 (2001).
51. S. F. Gabriel Krens, J. H. Veldhuis, V. Barone, D. Čapek, J.-L. Maître, G. W. Brodland, C.-P. Heisenberg, Interstitial fluid osmolarity modulates the action of differential tissue surface tension in progenitor cell segregation during gastrulation. *Development* **144**, 1798–1806 (2017).
52. R. Fernandez-Gonzalez, S. de Matos Simoes, J.-C. Röper, S. Eaton, J. A. Zallen, Myosin II dynamics are regulated by tension in intercalating cells. *Dev. Cell* **17**, 736–743 (2009).
53. R. Clément, B. Dehapiot, C. Collinet, T. Lecuit, P.-F. Lenne, Viscoelastic dissipation stabilizes cell shape changes during tissue morphogenesis. *Curr. Biol.* **27**, 3132–3142.e4 (2017).
54. V. Ferro, M. Chuai, D. McGloin, C. J. Weijer, Measurement of junctional tension in epithelial cells at the onset of primitive streak formation in the chick embryo via non-destructive optical manipulation. *Development* **147**, dev175109 (2020).
55. M. F. Staddon, K. E. Cavanaugh, E. M. Munro, M. L. Gardel, S. Banerjee, Mechanosensitive junction remodeling promotes robust epithelial morphogenesis. *Biophys. J.* **117**, 1739–1750 (2019).
56. T. Chung, *Computational Fluid Dynamics* (Cambridge Univ. Press, 2012).

57. J. S. Bois, F. Jülicher, S. W. Grill, Pattern formation in active fluids. *Phys. Rev. Lett.* **106**, 028103 (2011).
58. R. Sknepnek, I. Djafer-Cherif, M. Chuai, C. Weijer, S. Henkes, Generating active T1 transitions through mechanochemical feedback. *eLife* **12**, e79862 (2023).
59. G. Haller, Lagrangian coherent structures. *Annu. Rev. Fluid. Mech.* **47**, 137–162 (2015).
60. C. Cui, X. Yang, M. Chuai, J. A. Glazier, C. J. Weijer, Analysis of tissue flow patterns during primitive streak formation in the chick embryo. *Dev. Biol.* **284**, 37–47 (2005).
61. J. Firmino, D. Rocancourt, M. Saadaoui, C. Moreau, J. Gros, Cell division drives epithelial cell rearrangements during gastrulation in chick. *Dev. Cell* **36**, 249–261 (2016).
62. J. Ranft, M. Basan, J. Elgeti, J. F. Joanny, J. Prost, F. Jülicher, Fluidization of tissues by cell division and apoptosis. *Proc. Natl. Acad. Sci. U. S. A.* **107**, 20863–20868 (2010).
63. S. B. Shah, I. Skromne, C. R. Hume, D. S. Kessler, K. J. Lee, C. D. Stern, J. Dodd, Misexpression of chick Vg1 in the marginal zone induces primitive streak formation. *Development* **124**, 5127–5138 (1997).
